# Evaluation of antenatal point-of-care ultrasound training workshops for rural/remote healthcare clinicians: a prospective single cohort study

**DOI:** 10.1186/s12909-022-03888-5

**Published:** 2022-12-30

**Authors:** Amber Bidner, Eva Bezak, Nayana Parange

**Affiliations:** 1 Allied Health and Human Performance, The University of South Australia, Corner of North Terrace and Frome Road, GPO Box 2471, Adelaide, SA 5001 Australia; 2 Department of Physics, The University of Adelaide, North Terrace, Adelaide, SA 5001 Australia

**Keywords:** Medical education/training, Obstetrics and gynaecology, Ultrasound (US), Sonography, Point-of-care- ultrasound (PoCUS), Antenatal, Training, Rural, Remote, Low-resource setting

## Abstract

**Background:**

There is limited access to life-saving antenatal ultrasound in low-resource rural and remote settings worldwide, including Australia, mainly due to shortages in skilled staff. Point-of-care ultrasound (PoCUS) offers a viable solution to this service deficit, however, rural clinicians face many barriers accessing training and professional development critical to advancing their clinical practice. Standards for PoCUS training and competency assessment are unclear. Regulation is lacking globally, allowing untrained and inexperienced clinicians to practice PoCUS clinically.

**Methods:**

This prospective single cohort study aimed to evaluate antenatal PoCUS training workshops for General Practitioners (GPs) and Midwives/Nurses (M/Ns) from rural/remote Australia, assessing the impact of the training on trainees’ knowledge, confidence and translation of PoCUS into clinical practice. Two-day antenatal ultrasound workshops were delivered at the University of South Australia (UniSA) in 2018 and 2019 to 41 rural/remote clinicians . The training was designed and evaluated using the New world Kirkpatrick Evaluation Framework. Sixteen GPs and 25 M/Ns with mixed prior ultrasound experience were funded to attend. The course consisted of lectures interspaced with hands-on training sessions using high-fidelity simulators and live pregnant models. Pre- and post-knowledge assessments were performed. Post-workshop evaluation and follow-up surveys (3- and 6-month post-training) assessed the workshops and changes to trainees’ clinical practice. A 2-day follow-up training session was conducted 12 months after the workshops for 9 trainees.

**Results:**

Pre/post knowledge testing demonstrated a 22% mean score improvement (95% CI 17.1 to 27.8, *P* < 0.0001). At 6 months, 62% of trainees were performing PoCUS that had assisted in patient management and clinical diagnosis, and 46% reported earlier diagnosis and changes to patient management. 74% of trainees had increased scanning frequency and 93% reported improved scanning confidence.

**Conclusion:**

This study demonstrated intensive 2-day workshops can equip clinicians with valuable antenatal PoCUS skills, offering a viable solution to assist in the assessment and management of pregnant women in the rural/resource-poor setting where access to ultrasound services is limited or non-existent. Geographical isolation and lack of onsite specialist supervision poses an ongoing challenge to the continuing professional development of remote trainees and the implementation of PoCUS.

**Supplementary Information:**

The online version contains supplementary material available at 10.1186/s12909-022-03888-5.

## Background

Since the first ultrasound images of the fetus were published in the Lancet in 1958 [1], ultrasound has advanced to become the primary imaging modality in pregnancy [2–5]. In addition to estimating due dates, monitoring fetal growth and well-being, detecting anomalies and guiding specialist referral, antenatal ultrasound can facilitate the early detection of life-threatening complications such as ectopic pregnancy, fetal malpresentation, multiple pregnancies, placenta praevia and placental abruption [4, 6–8]. The International Society of Ultrasound in Obstetrics and Gynecology (ISUOG) have published guidelines recommending women receive two antenatal ultrasound examinations during a normal low-risk pregnancy [3, 9, 10], and The World Health Organization (WHO) recommend one antenatal ultrasound before 24 weeks of pregnancy [6]. However, studies into service accessibility in low-resource settings and rural regions throughout the world, where higher maternal and fetal mortality rates are reported [11–14], indicate women are not receiving this recommended care [15–17]. Over 90% of maternal deaths worldwide are estimated to occur in low-resource settings, and most are considered to be preventable [13, 14]. There is a global shortage of trained sonographers in these regions. Skills shortages have been reported in rural Australia for over a decade, where many remote medical centres have no onsite sonographer and rely on visiting professionals available as infrequently as one day per month [15, 18, 19]. Years of study and training are required to produce qualified sonographers in Australia. Available courses, most university conferred, are expensive and include a clinical practice component of which there are insufficient and declining placement opportunities [18, 20, 21]. Once trained it is challenging to entice professionals to relocate and remain in rural locations [18, 20, 21]. Upskilling the rural workforce in antenatal Point-of-Care ultrasound (PoCUS) is a viable solution to assist with this deficit and can offer substantial benefits to these under-resourced communities [22].

Defined as ultrasound imaging performed and interpreted by the healthcare provider at the bedside, PoCUS provides targeted scans to assist procedures or direct care and specialist referal [23]. Within the field of obstetrics, PoCUS can assist clinicians in the accurate estimation of due dates and early detection of potentially life-threatening complications, allowing for appropriate and timely pregnancy care planning and referral, which is crucial for remotely located women who may need days of travel to access specialist obstetric services [7, 15, 24]. Antenatal PoCUS does not replace formal ultrasound imaging performed by a trained sonographer. The Australian Department of Health (ADH) recommends a pre-planned schedule of antenatal visits, including up to four formal ultrasound examinations for a healthy low-risk pregnancy (8–14 week ultrasound to determine gestational age, 11–14 week fetal anomaly/nuchal translucency screening ultrasound, 18–20 week morphology ultrasound, and 36 week ultrasound for suspected non-cephalic presentation). In Australia, for women with access, these are performed most often in an imaging department by a trained sonographer using high-resolution ultrasound equipment and reported by a qualified radiologist or sonologist. Unlike formal ultrasound imaging, PoCUS can be performed anywhere by any healthcare worker, often using portable ultrasound equipment. While capable of producing high-quality images, this equipment does not provide the resolution and quality of a standalone ultrasound unit from an imaging department. However, their affordability and advantage of portability has helped establish PoCUS in many medical fields [2, 25].

Published literature and our research has shown many rural women do not present for antenatal care or ultrasound imaging until late in pregnancy or not at all [26–28]. A strength of PoCUS in the rural and low-resource context comes from its being, in some cases, the only imaging option available. For example, a rural clinician being able to establish gestational age in the first-trimester (when it is most accurately estimated) using PoCUS can allow for accurate timing of formal ultrasound imaging and scheduling of pregnancy care. Gestational age and estimated due date can also assist in detecting fetal growth disorders such as intrauterine growth restriction and macrosomia, and lead to confident identification of pre-and post-term labour critical to remote patients who may require considerable time and logistical planning for an assisted delivery. High-risk pregnancies (e.g. multiple pregnancy, placenta previa) can also be identified by rural clinicians using PoCUS, allowing for increased monitoring, triage and timely referral for advanced pregnancy management.

Despite the advantages offered by PoCUS, access to an ultrasound machine is insufficient for reliable service delivery; as a highly-skilled, operator-dependent modality, ultrasound requires appropriate and ongoing training of experienced healthcare professionals for safe and effective implementation into clinical practice. The WHO advocates all countries adopt a standardised curriculum and competency assessment for teaching PoCUS to improve the safety and quality of antenatal services and obstetric care [6]. However, training and competency assessment guidelines remain varied [29], and in many countries, including Australia, healthcare workers may perform PoCUS with little or no training, experience or formal accreditation. This lack of regulation may allow inexperienced and untrained practice, which represents a potential risk to patients and ensuing financial burden to healthcare systems in cases of misdiagnoses and unnecessary specialist referrals or patient transfers [18, 29–32]. However, given the urgent need for these skills and the difficulty accessing training in remote areas, regulation should be implemented carefully to preserve the time and financial advantages offered by PoCUS training.

The only certified pathway for PoCUS in Australia is through a ‘Certificate in Allied Health Performed Ultrasound’ (CAHPU) [33] or for doctors a ‘Certificate in Clinician Performed Ultrasound’ (CCPU) [34] which are managed by the Australasian Society for Ultrasound in Medicine (ASUM). This accreditation is not required to practice but is necessary to claim remuneration for PoCUS scanning performed in rural/remote areas through the government’s Medicare Benefits Schedule. Rural clinicians face additional barriers accessing this accreditation pathway which stipulates attendance at approved training courses (mostly city-based) and a proportion of required assessments be performed under direct supervision of an expert with educational feedback provided.

Published literature on Antenatal PoCUS training report generally positive findings, supporting the upskilling of health professionals in low-resource settings [35]. However, widely varying training and competency assessment methods are described and study quality is mixed. Only 1 Australian study on antenatal PoCUS training has been published in the last decade [36]. Our preliminary research included a national survey on ultrasound access and use in rural and remote Australia, which identified a lack of trained staff and inaccessibility of ultrasound equipment as key barriers to PoCUS in these communities. Rural clinicians face many obstacles to accessing training opportunities needed to safely perform PoCUS, including geographical isolation (distance from training courses), heavy clinical caseloads and lack of locum staff to cover absences to attend training [37, 38]. In response to this service and skills deficit, this mixed methods prospective pilot study (The Healthy Newborn Project- HNP) was designed to deliver 2-day antenatal PoCUS training workshops to rural and remote Australian Midwives/Nurses (M/Ns) and General Practitioners (GPs). This paper describes the delivery and evaluation of the workshop model. It investigates the impact on trainees’ knowledge and confidence, and the translation of PoCUS into clinical practice (PoCUS use and indications). Challenges to workshop delivery and barriers to training and PoCUS use are described with potential solutions. Findings may make a valuable contribution to obstetric PoCUS education in the rural/low-resource setting, and help guide curriculum development and health policy to increase the uptake and safe integration of this vital skill by rural healthcare workers, and improving healthcare services for rural women.

## Methods

**Fig. 1:**
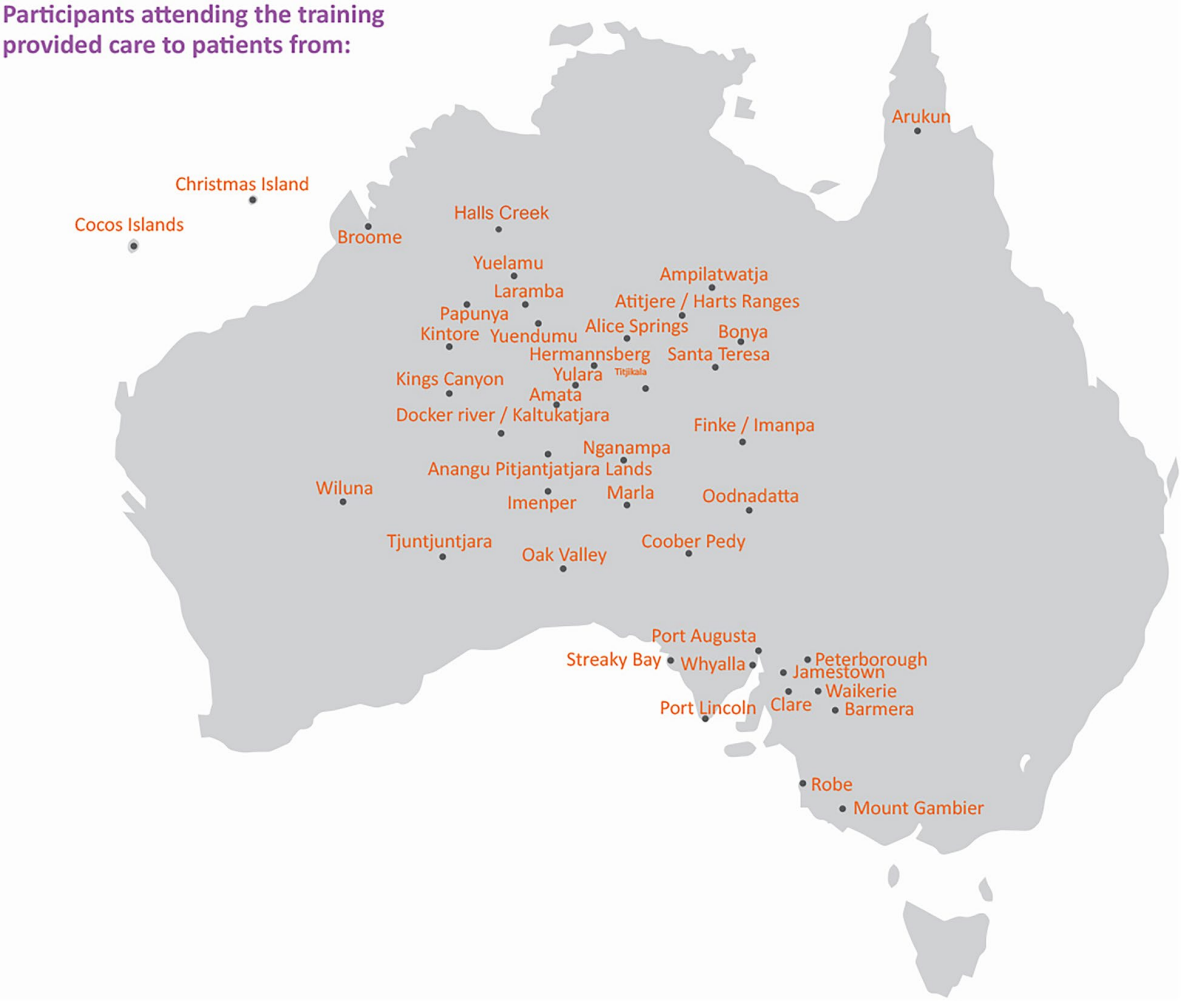
Clinic locations of trainees (GPs, M/Ns) attending antenatal PoCUS training workshops

Two-day antenatal PoCUS training was provided in 2018 and 2019 to a convenience sample of 25 Midwives/Nurses (M/Ns) and 16 General Practitioners (GPs) at the University of South Australia’s (UniSA) Adelaide city campus in a simulated ultrasound laboratory. Eligible trainees were working in rural/remote areas (see Fig. 1 for clinic locations), providing care to antenatal patients with access to ultrasound equipment. No prerequisite ultrasound training or experience was required. Outreach funding from The Hospital Research Foundation (THRF) paid participant travel, accommodation and training costs.

The workshop’s design and content were based on years of experience delivering PoCUS workshops in under-resourced regions in Australia and developing countries, and have been adapted to suit the Australian rural context. The training model aligns with ASUM's ‘Guide to providing an ultrasound workshop’ [39] and antenatal PoCUS syllabus [33, 34]. Additional Table 1 [39] of supplementary material summarises these workshop requirements, and Additional Table 2 [40, 41] lists methods for PoCUS competency assessment.

**Table 1:** Course content and practical skills checklist

Course content	Practical skills checklist		
**Two-day training schedule**	**Instrumentation**	**First-trimester**	**Second / Third-trimester**
**DAY 1**	- Power	- Fetal heart trace	- Fetal heart trace / M-Mode
- Acknowledgment to country and introductions			
- Course requirements/objectives, materials, practical skills checklist	- Time gain compensation (TGC)	- Gestational sac	- Fetal presentation
- Pre-course baseline knowledge assessment		- Yolk sac	
**Lecture 1**- Basic scanning principles, transducer manipulation, transducer and image orientation, knobology and ergonomics	- Gain	- Crown to rump length (CRL)	- Amniotic Fluid Volume
**Lecture 2**- Fetal lie, placental position and amniotic fluid volume (AFV)- Maximum Vertical Pocket	- Depth		
**Practical session**- 2 simulator stations and 2–3 pregnant volunteer stations	- Zoom	- Adnexae	- Placenta
**Lecture 3**- First-trimester ultrasound and pregnancy dating		- Free fluid	- Estimated due date
**Lecture 4**- Fetal viability & cardiac M-mode assessment	- Focus		
**Lecture 5**- Second and third-trimester biometry-			
- Scanning requirements	- Calliper use		Biometry: - BPD - HC - AC - FL
- Measurements-Biparietal Diameter (BPD), Head circumference (HC), Abdominal circumference (AC), Femur length (FL)	- Annotation		
- Measurement interpretation	- Image storage		
**Practical session**- 2 simulator stations and 2–3 pregnant volunteer stations			
**DAY 2**			
**Lecture 6**- Miscarriage, pregnancy of unknown location, ectopic pregnancy			
**Lecture 7**- Multiple pregnancy			
**Practical session**- 2 simulator stations and 2–3 pregnant volunteer stations			
**Lecture 8**- Communication and documentation- report writing and medicolegal considerations			
**Practical session**- 2 simulator stations and 2–3 pregnant volunteer stations			
- Post-course knowledge assessment			
- Anonymous post-course training evaluation			

Faculty-to-trainee ratio during practical sessions ranged from 1:2 to 1:4 with periods of 1:1 supervised practical instruction and assessment. A 1:3 ratio or less of trainees-to-ultrasound workstations was maintained for all 4 workshops

**Table 2:** Workshop Participants- number, role, previous ultrasound experience/training

**Workshop**	**GPs**	**M/Ns**	**Total**	**Previous ultrasound training/experience**
Workshop 1	6	6	12	9/12 (75%)
Workshop 2	4	9	13	7/13 (54%)
Workshop 3	6	10	16	11/16 (69%)
**Total all 3 workshops**	**16**	**25**	**41**	**27/41 (66%)**
Follow-up/Refresher Workshop 4	1	8	9	All attended one of the initial workshops 1–3

Three workshops provided training to a total of 41 participants in groups of 12–16. Participants with varied previous ultrasound experience were trained together in multidisciplinary groups. Instructors were accredited sonographers with 3 to 14 years experience teaching in UniSA’s Postgraduate Diploma in Medical Sonography; one instructor had specialist obstetrics and gynaecology medical training and extensive experience delivering outreach PoCUS workshops in rural Australia and developing countries. Pre-reading resources were sent several weeks in advance of the workshops. The training and material provided at the 3 initial workshops were identical and delivered by the same facilitators. Didactic sessions (7 hours total) were interspaced with the practical sessions (6 h total), allowing demonstration on the equipment following theoretical content delivery and immediate practice of techniques. Course content and practical skills covered during training and trainee-to-faculty ratios are outlined in Table 1.

CAE Vimedix OB-GYN high-fidelity simulators [42] (mannequin, high-definition monitor, dedicated computer and simulated transducer) were used initially for learning probe manipulation and manual scanning technique. Live pregnant models (12 per workshop) of varying second and third and 3-trimester gestations were also used for practical training. Only healthy women with low-risk pregnancies and prior normal first-trimester formal ultrasounds were eligible to volunteer, each limited to a total of 30 min scanning time. Sonosite Edge-II and Sonosite M-Turbo portable units and Phillips iU22xMatrix standalone ultrasound units were used in practical sessions. Participants were encouraged to bring their own portable ultrasound equipment for training.

### Assessment and evaluation

A pre-workshop survey was performed to collect trainee demographics, including clinical role, years of experience, and prior PoCUS training and scanning experience. This preliminary survey also explored current use and indications for PoCUS, and areas of antenatal PoCUS training theclinicians felt would benefit their practice to assist in curriculum design. Identical pre- and post-training knowledge assessment was conducted on ultrasound principles/physics, image optimisation, biometry measurements, obstetric anatomy and pathology, patient communication and image review. Anonymous post-workshop evaluations (Post-workshop evaluation form available in supplementary material) were completed by all trainees immediately following training to rate course content (theoretical and practical), design, instructors, and highlight areas for improvement. Online follow-up surveys were conducted at 3- and 6-months (6-month follow-up survey form available in supplementary material) following the workshops to investigate scanning application and frequency of use, self-reported confidence, change in clinical practice/behaviour and the clinician’s perception of impact on patient outcomes.

All surveys were developed by a multidisciplinary research team and included a mix of multiple choice, multiple response, Likert scale and free-text formats. The immediate post-workshop evaluation and 3- and 6-month follow-up surveys were adapted from forms used by UniSA’s medical sonography program to evaluate workshop design, content, presenter and impact. These established evaluation tools have been in use since 2008, with all validity and reliability tests performed at that time. Content validation was performed by healthcare professionals external to the research project. Individual trainee responses collated from the pre-workshop, 3- and 6-month surveys were compared for reliability and internal consistency.

### Follow-up training and support

**Fig. 2:**
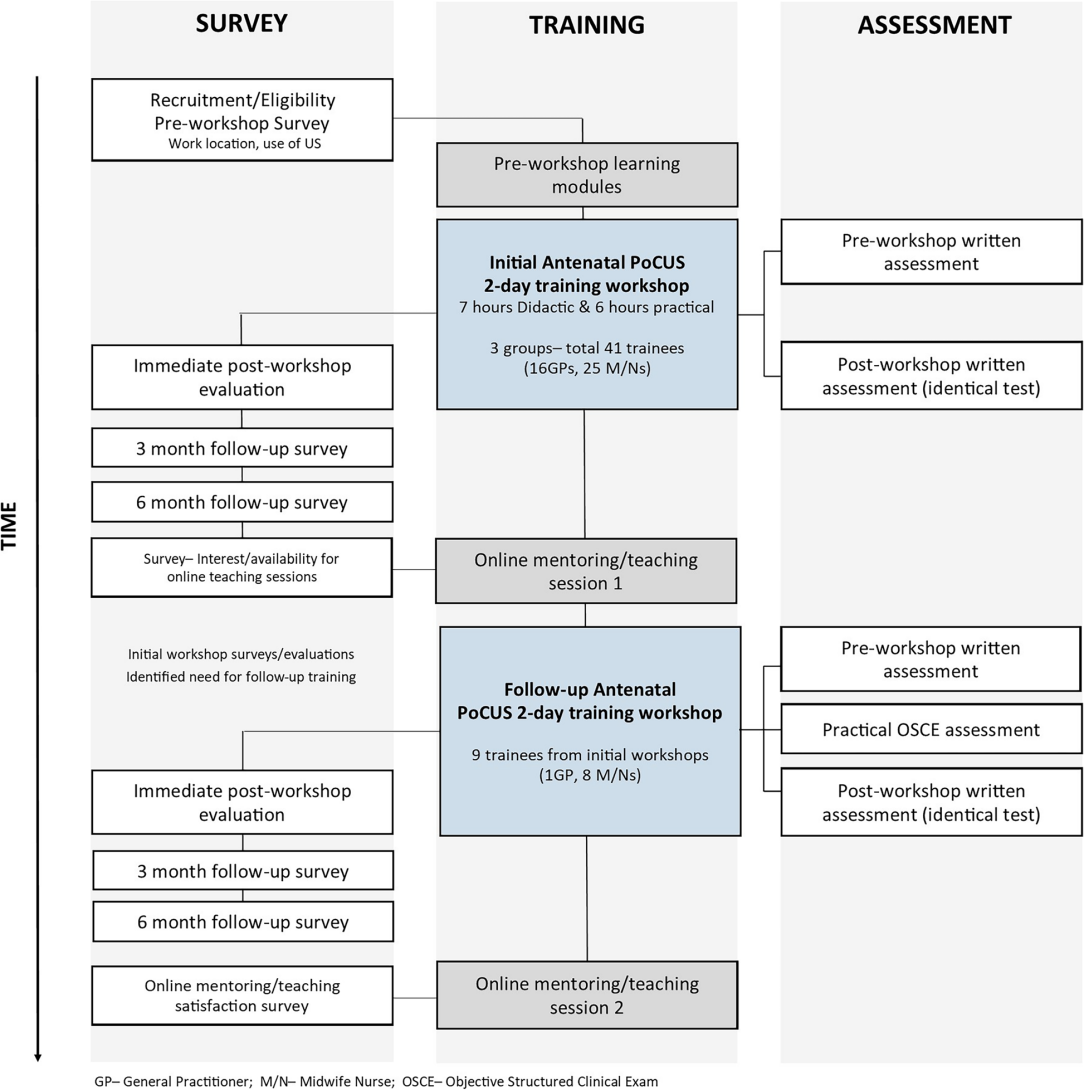
Flow chart of antenatal PoCUS workshops- Training, assessment and surveys

Two online group mentoring/teaching sessions were provided 5 to 7 months after the training sessions, and an online forum was setup for trainees to network and access all course materials, which were also provided on USB. A 2-day follow-up training workshop was held 12 months after the initial training workshops for 9 trainees who were provided with the opportunity to request teaching content. This workshop reviewed important concepts building on the trainees’ previous knowledge and skills, explored case studies/images, and dedicated more time to practical training. Learning was evaluated using an identical pre- and post-course knowledge test, and a post-course Objective Structured Clinical Exam (OSCE) practical assessment was administered (First and Second-trimester OSCE assessment forms available in supplementary materials 5 and 6). All assessments were adapted from validated tests used in the University’s sonography teaching program. First-trimester OSCE assessment was performed on high-fidelity simulator and second-trimester on live models. Each case-based 6–7 part examination assessed patient communication, scan technique and accuracy of measurements with an overall ‘satisfactory’ or ‘not satisfactory’ grading. Anonymous post-workshop evaluation and 3- and 6-month follow-up surveys were conducted. Figure 2 provides a flowchart of antenatal PoCUS training, assessments and surveys conducted.

### Analysis

Microsoft Excel 16.0 and IBM SPSS statistics 27 were used to generate data tables and analyse means and frequencies. Paired t-test analysis of trainees’ pre- and post-test results was performed with stratification by profession/role and previous training/experience, and graphs presented using Graphpad Prism 8 software. Qualitative data was compiled using Excel spreadsheets, and a thematic analysis performed using Nvivo (2020) to explore the qualitative responses and guide a narrative review of concepts. The PoCUS workshops were appraised using the New World Kirkpatrick Evaluation Framework (NWKEF), an updated version of an established outcome-focused model for clinical education assessment [43–45]. Additional Fig. 1 of supplementary material shows the HNP workshops against the NWKEF.

Endorsement for the workshops was received from ASUM and ISUOG, with ethics approval from UniSA’s Human Research Ethics Committee (Project ref: 201,543). This study and manuscript followed the equator network ‘Strengthening the Reporting of Observational Studies in Epidemiology’ (STROBE) guidelines for reporting observational studies [46].

## Results

Table 2 details each workshop group, including trainee profession/role and percentage with previous ultrasound training/experience. Detailed trainee demographics are provided in Additional Table 3 of supplementary material.

One respondent who only attended 1 day of training and failed to complete the 3-month survey was removed from all post-training analyses. Responses from 2 other trainees, one who did not complete the 3-month survey and one who did not complete the 6-month survey, were removed from the frequency of ultrasound use analysis.

### Pre-workshop survey

The pre-workshop survey indicated 44% (18/41) of all participants were performing ultrasound clinically before the training (1–5/week, average 2/week). Indications for PoCUS use reported in order of frequency were: estimating gestation, referral for care, determining fetal presentation, assessing fetal viability, fetal growth assessment, PV bleeding, confirmation of pregnancy and patient request. Previous training programs had been attended by 54% (22/41) of participants, and 63% (26/41) reported having used ultrasound clinically in the past. Only 3 participants (7%) were performing ultrasound clinically with no prior formal training, and 7 (17%) had completed training but were not currently performing ultrasound at their clinic. Ninety percent (37/41) of trainees had patients who were required to travel out of their community for ultrasound services, travelling between 1 to 8 hours (Average 3.8 h) and distances of 70 to 3000 kilometers, some requiring flights and 1–2 nights’ accommodation away from home and family.

### Pre-post course knowledge assessment

**Fig. 3:**
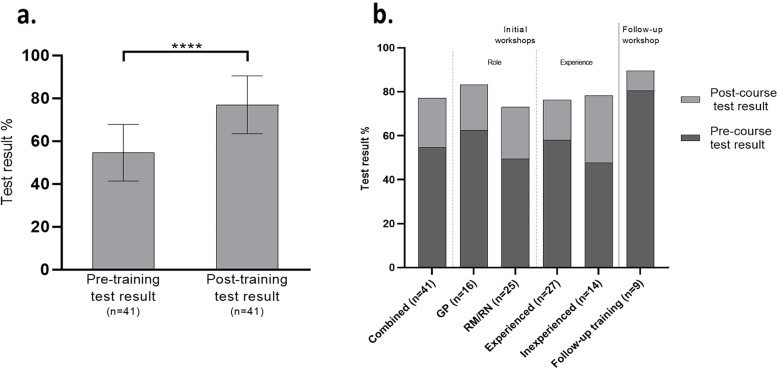
**a** Pre- and post-test results for initial training workshops (*n* = 41) showing significant improvement in mean test scores (*p* < 0.001). **b** Pre-and post-test results stratified by trainee role and previous ultrasound experience

Theoretical knowledge assessment performed before and after training demonstrated significant improvement in mean test scores of 22.4% for the initial workshops *(p* < 0.001). Both pre-and post-test scores were higher for GPs, but greater improvement was seen in the M/Ns cohort. Those with no prior ultrasound experience had the lowest pre-test scores but showed the greatest improvement overall (30.4%). Figure 3 a and b display trainees’ mean pre-and post-test results with stratification by role and previous ultrasound experience/training. Additional Table 4 of supplementary material provides the mean test results, with confidence intervals and *p*-values, of all workshops and stratified groups.

### Follow-up workshop practical OSCE assessment

For participants (9) who attended the follow-up training session (Workshop 4), pre-and post-test scores were significantly higher compared to the initial workshops (*p* < 0.008), demonstrating retention of knowledge and an increased understanding of concepts following clinical scanning experience, but showed the smallest improvement between tests (9.2%). Of the 9 trainees, 2 were unable to satisfactorily complete the first-trimester OSCE. Second-trimester OSCE assessment proved more difficult for trainees, with 5 failing to perform at a satisfactory level. This may reflect the more complex requirements for second-trimester scanning and the complexity of scanning real patients over the simulated (Vimedix) first-trimester OSCE.

### Post-workshop evaluation

All trainees found the training valuable and relevant to their role, highly rating the workshop content, design, activities and facilitators, and indicated increased scanning confidence after learning. Trainees either strongly agreed or agreed that the difficulty level was appropriate and they would be able to utilise what they had learnt in clinical practice. The Vimedix simulators and live pregnant models were reported as the most valuable aspect of the training by 95% (39/41) of participants.

### 3- and 6- month post-workshop survey

**Fig. 4:**
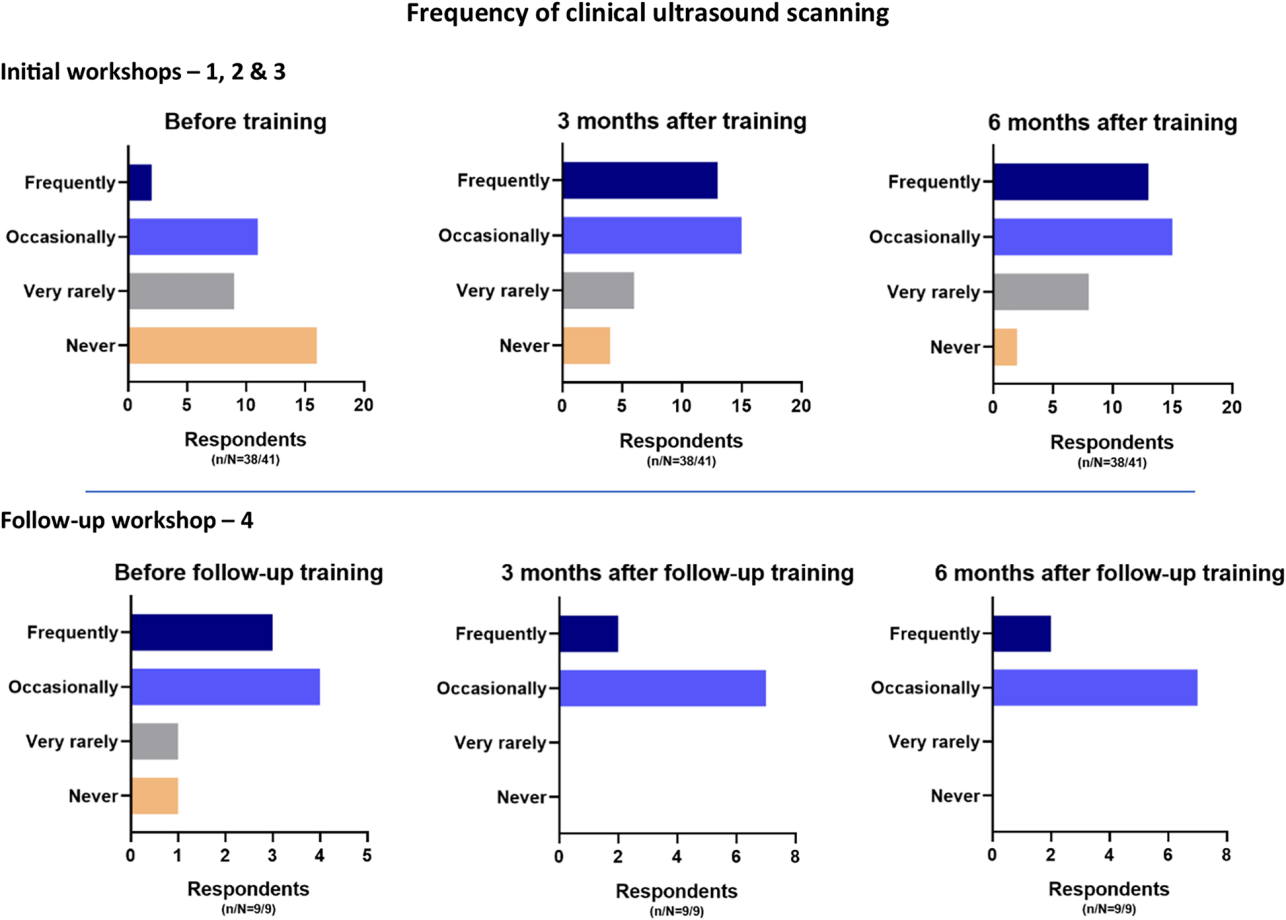
Frequency of ultrasound scanning before training and at 3- and 6-months after training.*3 respondents censored due to incomplete data (3 or 6 month survey not completed)

At 3 months, 11% (4/38) of participants were not performing PoCUS, and 5% (2/38) were not scanning at 6 months. Reasons provided for never or rarely using PoCUS following training included: broken equipment, sub-optimal equipment, no/few antenatal patients seen clinically, and working mainly non-clinically in management/administration. Scanning frequency before training and at 3 and 6 months post-training for initial and follow-up workshops are provided in Fig. 4.

At 6 months after the workshop, trainee/self-reported data indicated 87% (34/39) of trainees had applied knowledge gained from the training to clinical situations, and 74% (29/39) reported having observed an impact on patient outcomes. As a result of attending the workshop, 62% (24/39) reported performing scans that had assisted in patient management and diagnosis, with earlier diagnosis and changes to patient management reported by 46% (18/39) of trainees. The main change to trainees’ clinical practice/behaviour reported by 69% (27/39) of trainees was increased confidence in skills and ability to perform antenatal PoCUS. Additional Table 5 of supplementary material reports areas of improved confidence and practical skill by frequency of trainee response. Practise and follow-up training was named by 78% (28/36) of trainees as necessary to consolidate skills and further increase confidence and frequency of PoCUS use.

## Discussion

This prospective single cohort study of 41 rural/remote clinicians demonstrated a statically significant improvement in knowledge-based test scores following the delivery of 2-day antenatal PoCUS training workshops in Adelaide, Australia. Trainees attending a second round of refresher training scored higher on pre-and post-test scores demonstrating retention of knowledge and increased understanding of concepts following 12 months of clinical scanning experience. The quantitative test data demonstrated the greatest test score improvements in Midwives/Nurses (M/Ns) and the ultrasound inexperienced cohorts. This result may reflect the fact that 81% (13/16) of the General Practitioners (GPs) had prior ultrasound training and experience (higher pre-course baseline knowledge) compared to 56% (14/25) of M/Ns. This trend is reflected in the literature, with ultrasound training being increasingly incorporated into undergraduate medical curricula and on-the-job training [47, 48], but less established in non-physician (nursing and midwifery) education programs and scope of practice [49, 50]. In most developing countries and low-resource settings, antenatal care is provided primarily by midwives and nursing staff, which presents an opportunity upskill these essential workers [48, 51–55].

Course evaluation and later follow-up showed trainees found the program relevant to their roles in rural health, and either ‘agreed’ or ‘strongly agreed’ that the difficulty level was appropriate and they would use what they had learnt in their clinical practice. Workshop content, design, activities, and facilitators were highly rated, with the Vimedix simulators and live pregnant models reported by 95% of participants as the most valuable aspect of the training. This supports the benefits of designing a program with an emphasis on practical hands-on training, which the program interspaced with theoretical content to consolidate learning and transfer theory into practice. Increased confidence, scanning frequency and impact on patient outcomes and management (as perceived by the clinicians) were described by the trainees as a result of the training; improvement in patient management and clinical diagnoses was reported by 62% of trainees, and earlier diagnoses and changes to patient management was reported by 46% of trainees. Other studies evaluating obstetric PoCUS training report similar improvements in pre-post course testing [56–59], trainee confidence [60–63], and knowledge retention [36, 56, 64].

The existing body of evidence evaluating antenatal PoCUS training stems largely from developing resource-poor countries. However, parallels in service access and health outcomes can be drawn between developing countries and rural and remote regions in developed nations [65, 66]. These studies commonly suffer limitations inherent to research conducted in remote settings, including low participant numbers, convenience samples, and loss to follow-up [32]. Our own systematic review [22] of PoCUS training evaluations in published literature highlighted a lack of comparable high-quality studies needed to establish a stronger evidence base for antenatal PoCUS. However, findings were generally positive, with improved knowledge and competence being reported despite the varying course durations (3 h to spanning several years). Variation in competence assessment and duration of trainee follow-up was also observed, with 11 of the 27 identified studies not surpassing the Kirkpatrick Evaluation Framework levels 1 or 2 which assess immediate reaction to training and knowledge gained. Almost half the studies investigated patient outcomes (KEF Level 4), several going further to ascertain if PoCUS changed the patient diagnosis and if this impacted their management, providing the most robust evidence for the impact of PoCUS. Kolbe et al. [48] found 52% of patients had a new diagnosis following antenatal PoCUS, of which 48% led to a change in patient management. In Rominger et al. [55] 34% of patients had a PoCUS directed change in diagnosis, with 78% leading to a modification of clinical management.

### Multidisciplinary mixed experience groups

Delivering a training program to meet individual learning needs in a multidisciplinary mixed experience group, like those trained in this study, is challenging. The workshop evaluations revealed some opposing opinions on course content. For example, one trainee felt *“Basic physics- frequency of sound waves *etc*.” w*as the least valuable content of the workshop, while another stated they wanted *“to learn more about basic physics”*. The scope of practice between M/Ns and GPs and those with prior training compared to novice practitioners may be reflected in the comments from some trainees who appreciated the coverage of ectopic pregnancy and requested more detail in future workshops, while others found this content to be beyond their scope of practice. Running basic and advanced workshops separately was considered during the workshops design, however recruiting sufficient numbers of similarly experienced trainees with the same availability was not feasible. Allocating trainees to separate sub-groups with curricula and objectives catered to their experience and skill level could mitigate this problem but may exclude the advantages of interdisciplinary collaboration and learning [49]. This approach is also difficult to accomplish when delivering didactic content to small groups with a single trainer common to remote settings. A ‘flipped classroom’ pedological approach through the provision of pre-reading materials and learning modules, as used in this study, is an effective technique for teaching task-based skills like PoCUS [49, 67, 68], helping minimise the knowledge gap between experienced and inexperienced trainees and enabling faster transition through basic concepts. Preliminary knowledge testing prior to course entry to ensure understanding of assigned pre-course learning was not performed but has been reported in the literature [49, 51, 52, 59, 69].

### Simulators and models

In this study, first-trimester ultrasound and clinical pathology that were unavailable for demonstration on live models were instead demonstrated using high-fidelity simulators, which provided a safe, patient-free learning environment, and were highly rated/commended by the training participants. Ideally, training should utilise both simulated and real-life patients with strictly limited scanning times for pregnant volunteers and heavier reliance on phantom models and virtual/simulation technologies in early training [70–72] as recommended by the ISUOG [41]. This reduces reliance on pregnant volunteers and provides the opportunity to scan simulated first-trimester pregnancies, often lacking in training courses due to the early gestation of the fetus and associated risk of identifying an unexpected abnormality in volunteers who are yet to receive formal scanning. A limitation of the simulators highlighted in feedback was the lack of controls (e.g. gain and focus) representative of an actual ultrasound unit. Other disadvantages of high-fidelity simulated technologies include the absence of the complexities of fetal movement and patient interaction, as well as the cost of implementing such systems, which could be prohibitive in low-resource settings [71, 73, 74]. Many trainees stated being able to train on their own equipment and become more familiar with the controls would have been beneficial, and despite being encouraged to bring their portable ultrasound units to the workshop, only 2 trainees were able due to active clinical use of the equipment and transport/insurance concerns.

### Change in practice and patient outcomes

Seventy-four percent of trainees reported an impact on patient outcomes resulting from their training (as perceived by the clinician), demonstrating the practical benefits of the 2-day workshop. Qualitative responses indicated this stemmed from their ability to: better plan pregnancy care and refer where appropriate, increase patient engagement and antenatal care compliance, more accurately estimate gestation early in pregnancies, provide reassurance and patient education, and reduce unnecessary travel.

Identifying high-risk pregnancies in remote settings, where co-morbidities (e.g. obesity, diabetes, substance abuse) are more prevalent [75, 76], is important for antenatal care planning. This includes frequent monitoring, specialist referral and establishing the potential need for pre-term-delivery; a logistical challenge for remotely located women. One Trainee reported *“I have been able to identify twin fetal heart activity in first trimester confirming pregnancy and increasing ANC visits accordingly… I have had an obese diabetic pregnant woman with a difficult abdominal palpation in remote community and was able to identify lie and position of fetus*.” Another *stated “Being able to provide anultrasoundat a woman’s first visit is hugely helpful in planning and coordinating care, especially when they have chronic diseases such as T2DM.”*

For some trainees, being able to offer PoCUS increased patient attendance at their clinic and provided earlier more accurate dating scans that are often missed in remote settings, and allowed for opportunistic scans on patients who would otherwise receive limited or no antenatal imaging throughout their pregnancy. Many instances of patients no longer needing to travel, some for days, to have a basic early dating scan where last menstrual period is unknown (required to plan antenatal care and schedule formal ultrasound imaging) were described. Gestational dating, most reliably assessed in the first-trimester, was the most frequent application for ultrasound use reported following training. Accurate estimation of due dates for delivery planning is crucial for remotely located women who may need days of travel to access obstetric care [7, 15, 24]. The early detection of problems and reduction of unnecessary travel/transfers can also provide direct economic benefits to healthcare systems. One respondent provided the example of a patient “*for whom it was not possible to find a fetal heart with the doppler unit. We were able to use the PoCUS unit to rapidly detect a fetal heart and live active fetus which saved a transfer, hospital resources and maternal anxiety*.”

A sample of qualitative responses on PoCUS application and patient impact (as perceived by the clinicians) with clinical examples are provided in supplementary material- Additional Table 6a: Application of learning/PoCUS to clinical practice and Additional Table 6b: Impact of training/PoCUS use on patient outcomes.

### Barriers to training and continuing professional development (CPD)

The most common barriers to accessing training and CPD opportunities raised by the trainees in order of frequency was: cost (course and travel); distance/remoteness; time for training; lack of relief staff; limited employer support; lack of onsite supervision/mentorship; availability and timing of courses; time to practise new skills with competing clinical duties/heavy workloads; no local/remote-setting courses available; poor internet access; and lack of credentialing opportunities. The training was scheduled on weekends due to lower staffing requirements at rural/remote clinics which often operate with minimal permanent staff and limited accessible relief staff [37]. Online mentoring and follow-up teaching was offered, with trainees surveyed to establish availability to accommodate work schedules. Many were unable to participate due to varying schedules, heavy clinical workloads and no or poor internet access (some conducting outback ‘bush’ visits). Of those who did attend (12), anonymous feedback was positive, with case study discussion and image review reported as the most helpful aspects by the majority, followed by networking with other clinicians.

Continuous access to a quality ultrasound machine was also reported as a barrier to trainees performing PoCUS upon return to work. In some cases, portable ultrasound machines were shared between numerous clinics and could be taken out to bush communities for days at a time. Several trainees reported equipment failure, one unable to scan due to the poor quality of their mobile ultrasound unit. While resources may be limited, a false economy may ensue where cheaper portable imaging equipment is purchased. A high-end portable ultrasound unit capable of providing high quality resolution can be purchased for around $40,000 (AUD). Cheaper units under $10,000 (AUD), including hand-held devices that can be adapted to a mobile phone or tablet, are available and the technology is improving but may not provide adequate resolution, particularly on obstetric patients with a higher Body Mass Index [77, 78].

Despite the above scheduling measures and coverage of travel and course costs by THRF, a theme which emerged from the trainees qualitative responses was the desire for onsite/workplace training to exclude the need to travel and reduce time away from work and family. A small number of the participants felt a lack of confidence scanning patients on returning to work due largely to lack of onsite supervision and assistance. To better address these identified barriers, the next round of HNP training provided remote-setting/onsite antenatal PoCUS workshops to 23 outback clinicians in Alice Springs in May and June of 2022. This format allowed the trainees to use and become more proficient on their own ultrasound equipment. Additional online group support/teaching sessions were held and access to trained sonographers for one-on-one consults and image review/feedback were offered to all past HNP training participants. Trainees were also encouraged to consider formal accreditation in PoCUS. The ASUM accreditation pathway provides clinicians with ongoing supervision and mentorship with longitudinal formative and summative assessment, and is the only means to claim remuneration through the Medicate Benefits Schedule in Australia. ASUM specifies half of the required assessments may be completed in a non-clinical environment, but under direct supervision with educational feedback provided. Teleultrasound may provide an alternative, allowing rural clinicians to be supervised and assessed by a distance city-based ultrasound expert. Making the accreditation pathway more accessible to rural clinicians could incentivise greater uptake of PoCUS training opportunities and better support clinicians practicing remotely.

While face-to-face teaching is optimal when learning a practical skill like US, online teaching and mentoring offers rural clinicians a means of support otherwise unavailable in their remote settings. Within our study, the use of a basic online meeting platform with the option of cursor control and screen sharing with trainees was well received. *“The online session was brilliant, I think it is very motivating to have sessions like that, it refreshed our memories. I particularly liked looking at the images and explaining what we were looking at…the ability to communicate by using the mouse at our end was great!”.* Advancements in Teleultrasound systems now make it possible for an experienced clinician/instructor to communicate from a distance via live video and text message with the trainee/clinician, view the ultrasound monitor, images and probe position, and even take control of the ultrasound machines functions, all in real time [79]. Augmented reality simulated technologies with similar capabilities are also emerging as a viable tool for distance education and support [80, 81]. While such systems require significant funding and infrastructure (quality internet), they do offer considerable advantages for remote supervision and support of trainees and may see greater utilisation in the future.

### Limitations

A small convenience sample of healthcare practitioners were recruited for the training. The total number of trainees was determined by available funding. While checklists of practical tasks were completed under instructor supervision, no formal practical assessment was conducted for the initial 3 workshop groups. At follow-up, 3- and 6-month surveys focused on trainees’ use/application of PoCUS, confidence, and changes in clinical practice behaviour, i.e. follow-up practical and knowledge assessments after a period of clinical scanning were not conducted for the initial three workshops (Workshops 1–3). Only the Follow-up/Refresher training group (Workshop 4) was assessed for practical scanning competence and learning retention at 12 months. Practical scanning improvement was unable to be measured/quantified for this group however as baseline practical assessment was not conducted at initial training. Several trainees were not scanning patients following training and 3 failed to complete either the 3 or 6 month survey.

Image quality on return to clinical practice was not evaluated. Expert image review is a useful measure for quality assurance and competence assessment, particularly where direct onsite supervision is not possible, as it may be performed asynchronously and remotely. Trainees were offered ongoing access to qualified trainers/sonographers and invited to send images for critique but no trainees took up this offer. While assessing actual patient outcomes would provide the most robust evidence of PoCUS impact, the scope of this pilot study and its limitations (funding and study duration) excluded the collection of patient clinical outcomedata. This study only assessed impact on patient management and outcomes from the clinician’s perspective.

### Future direction

The ultimate goal of the HNP is to establish an evidence-based, accessible and sustainable training program for rural clinicians. With extended funding, our future training iterations will see the delivery of antenatal PoCUS workshops directly to clinicians in their local communities (following the pilot training conducted in Outback Australia this year). We will also be working in consultation with ASUM towards making the formal accreditation pathway for PoCUS more accessible to rural clinicians in Australia.

Quality studies on antenatal PoCUS with a focus on patient outcomes data, longer-term competency assessment, trainee support/supervision on return to practice and economic utility are needed to provide more robust evidence of the value of PoCUS in the rural healthcare setting and its impact on patients and healthcare services. Researcher and clinical educators looking to implement or evaluate their own PoCUS courses should ensure attention is given to the challenges and barriers unique to rural and low-resource settings at all stages of development, implementation and evaluation.

## Conclusion

Intensive training workshops can equip clinicians with valuable skills and the confidence to perform PoCUS, presenting a viable solution to the ultrasound service access and skills shortage in rural and resource-poor settings. In this study, significant improvement in ultrasound knowledge and scanning confidence was evident following the delivery of 2-day antenatal PoCUS training workshops, with retention of knowledge demonstrated at 12 month follow-up assessment. Increased scanning frequency and changes to clinical behaviour impacting patient management and outcomes were described, with increased antenatal care attendance and compliance with care directives reported.

Interest in advancing skill-sets to take advantage of expanding technologies like ultrasound is evident amongst rural Australian clinicians. The future of healthcare and its education is moving towards a more cooperative interdisciplinary culture [82], providing the opportunity to use trainees’ unique experiences and individual strengths to enhance course design and foster collaborative practice. Providing clear objectives and varying curricula in breakout groups tailored to participants’ experience levels would benefit a multidisciplinary PoCUS training cohort. However, barriers to accessing training opportunities, as well as formal accreditation pathways and ultrasound equipment exist. Geographical isolation and lack of onsite expert supervision pose an ongoing challenge to the development of remote trainees’ PoCUS skills. Creative solutions for distance training and supervision are needed, such as those offered by telehealth technologies.

This paper may serve to guide educators of PoCUS in the development of training programs directed at rural practitioners, and inform policy for clearer standardised training and competency assessment guidelines needed to ensure safe clinical practice. Government initiatives to support rural clinicians accessing PoCUS training, equipment and formal accreditation is vital to strengthening workforce capacity in these under-resourced communities, and improving health outcomes for rural mothers and babies facing significant inequities in healthcare access.

## Supplementary Information

**Additional file 1: Table 1.** General workshop requirements defined by ASUM.

**Additional file 2: Table 2.** Methods for assessing PoCUS competency.

**Additional file 3.** Post-workshop evaluation form.

**Additional file 4.** 6-month follow-up survey form.

**Additional file 5.** First-trimester OSCE assessment form.

**Additional file 6.** Second-trimester OSCE assessment form.

**Additional file 7: Figure 1.** New World Kirkpatrick Evaluation Framework (NWKEF) [45] for training evaluation and the Healthy Newborn Project workshops.

**Additional file 8: Table 3.** Trainee demographics (role, clinical experience, clinic remoteness area, pre- and post-workshop test results, previous ultrasound experience and ultrasound unit used).

**Additional file 9: Table 4.** Pre-post test scores for all workshops, and initial workshops by position/role and previous ultrasound experience.

**Additional file 10: Table 5.** Areas of improved confidence and practical scanning.

**Additional file 11: Table 6a.** Application of learning/PoCUS to clinical practice: Qualitative responses from 3- and 6-month follow-up surveys. **Table 6b.** Impact of training/PoCUS use on patient outcomes: Qualitative responses from 3- and 6-month follow-up surveys.

## Data Availability

The datasets generated and/or analysed during the current study are available from the corresponding author on reasonable request. Additional data referred to but not included in the main manuscript is provided in supplementary material as ‘Additional Tables’ and ‘Additional Figures’.

## Abbreviations

AFV: Amniotic Fluid Volume
AC: Abdominal Circumference
ANC: Antenatal care
ASUM: Australasian Society for Ultrasound in Medicine’s
BPD: Biparietal Diameter
CPD: Continuing Professional Development
CRL: Crown to Rump Length
FL: Femur Length
GP: General Practitioners
HC: Head Circumference
ISUOG: International Society of Ultrasound in Obstetrics and Gynecology
M/N: Midwife/Nurse
NWKEF: New World Kirkpatrick Evaluation Framework
OSCE: Objective Structured Clinical Exam
PoCUS: Point-of-care ultrasound
PV: Per Vagina
STROBE: Strengthening the Reporting of Observational Studies in Epidemiology
T2DM: Type 2 Diabetes Mellitus
TGC: Time Gain Compensation
THRF: The Hospital Research Foundation
UniSA: University of South Australia
US: Ultrasound
WHO: World Health Organization

## References

[1] Donald I, Macvicar J, Brown TG (1958). Investigation of abdominal masses by pulsed ultrasound. Lancet (London, England).

[2] Campbell S (2013). A short history of sonography in obstetrics and gynaecology. Facts Views Vis Obgyn.

[3] Australian Department of Health (ADoH). Clinical Practice Guidelines: Pregnancy care. 2019th ed. Canberra: Australian Government; 2019. https://www.health.gov.au/sites/default/files/pregnancy-care-guidelines_0.pdf. Accessed 16 Jan 2022.

[4] Whitworth M, Bricker L, Mullan C (2015). Ultrasound for fetal assessment in early pregnancy. Cochrane Database Syst Rev.

[5] Nicolson M (2013). Imaging and Imagining the Fetus: The Development of Obstetric Ultrasound.

[6] World Health Organization (WHO) (2016). WHO recommendations on antenatal care for a positive pregnancy experience.

[7] Wiafe YA, Odoi AT, Dassah ET. Ultrasound Imaging - Medical Applications. In: Minin IVM, O.V., editor. Ultrasound Imaging - Medical Applications. London: IntechOpen; 2011. 10.5772/689.

[8] Murugan VA, Murphy BOS, Dupuis C, Goldstein A, Kim YH (2020). Role of ultrasound in the evaluation of first-trimester pregnancies in the acute setting. Ultrasonography (Seoul, Korea).

[9] Royal Australian and New Zealand College of Obstetricians and Gynaecologists (RANZCOG) (2016). Routine antenatal assessment in the absence of pregnancy complications RANZCOG.

[10] Salomon LJ, Alfirevic Z, Da Silva CF (2019). ISUOG Practice Guidelines: ultrasound assessment of fetal biometry and growth. Ultrasound Obstet Gynecol.

[11] Australian Institute of Health and Welfare (AIHW) (2019). Stillbirths and neonatal deaths in Australia 2015 and 2016.

[12] Australian Institute of Health and Welfare (AIHW) (2016). Maternal deaths in Australia 2016.

[13] World Health Organization (WHO). Maternal mortality: 19 September 2019. World Health Organization. 2019. https://www.who.int/news-room/fact-sheets/detail/maternal-mortality. Accessed 25 Mar 2020.

[14] World Health Organization (WHO). Newborns: improving survival and well-being: 19 September 2020. World Health Organization. 2019. https://www.who.int/news-room/fact-sheets/detail/newborns-reducing-mortality. Accessed 09 Apr 2021.

[15] Community Affairs References Committee (2018). Availability and accessibility of diagnostic imaging equipment around Australia.

[16] Hofmeyr GJ, Haws RA, Bergström S, et al. Obstetric care in low-resource settings: what, who, and how to overcome challenges to scale up? Int J Gynaecol Obstet. 2009;107 Suppl 1:S21-44, s−5. 10.1016/j.ijgo.2009.07.017. 10.1016/j.ijgo.2009.07.017 19815204

[17] McClure EM, Nathan RO, Saleem S (2014). First look: a cluster-randomized trial of ultrasound to improve pregnancy outcomes in low income country settings. BMC Pregnancy Childbirth.

[18] Australasian Society of Ultrasound in Medicine (ASUM) (2017). Minimum education & training requirements for ultrasound practitioners. Australas J Ultrasound Med..

[19] Stanton K, Mwanri L (2013). Global maternal and child health outcomes: the role of obstetric ultrasound in low resource settings. World J Prevent Med.

[20] Australian Sonographer Accreditation Registry (ASAR). Sonographer Accreditation. Australian Sonographer Accreditation Registry (ASAR). 2020. https://www.asar.com.au/sonographer-info/accredited-medical-sonographer/. Accessed 25 Mar 2021.

[21] Australian Sonographer Accreditation Registry (ASAR) (2020). The Australasian Sonographers Association 2019–20 Australian Government pre-budget submission.

[22] Bidner A, Bezak E, Parange N (2022). Evaluation of antenatal Point-of-Care Ultrasound (PoCUS) training: a systematic review. Med Educ Online.

[23] Australasian Society of Ultrasound in Medicine (ASUM). Discussion Paper: Definition of Point of Care Ultrasound (POCUS). The Australasian Society of Ultrasound in Medicine (ASUM). ND. https://www.asum.com.au/files/public/RealTime/2017/ASUM-Discussion-Paper-Definition-of-POCUS.PDF. Accessed 28 Mar 2021.

[24] Fentress M, Heyne TF, Barron KR, Jayasekera N (2018). Point-of-Care Ultrasound in Resource-Limited Settings: Common Applications. South Med J.

[25] Moore CL, Copel JA (2011). Point-of-care ultrasonography. N Engl J Med.

[26] Rumbold AR, Bailie RS, Si D (2011). Delivery of maternal health care in Indigenous primary care services: baseline data for an ongoing quality improvement initiative. BMC Pregnancy Childbirth.

[27] Australian Department of Health. Clinical Practice Guidelines: Pregnancy care. 2020th ed. Canberra: Australian Government; 2020. https://www.health.gov.au/sites/default/files/documents/2021/11/pregnancy-care-guidelines-pregnancy-care-guidelines.pdf. Accessed 16 Jan 2022.

[28] Australian Institute of Health Welfare (2021). Australia’s mothers and babies.

[29] Leonardi M, Murji A, D’Souza R (2018). Ultrasound curricula in obstetrics and gynecology training programs. Ultrasound Obstet Gynecol.

[30] Collins K, Collins C, Kothari A (2019). Point-of-care ultrasound in obstetrics. Australas J Ultrasound Med.

[31] Noriega O, Ho H, Wright J (2014). The Application of Hand-Held Ultrasound Scanner in Teaching of Telemedicine and Rural Medicine. Donald School J Ultrasound Obstet Gynecol.

[32] Rajamani A, Shetty K, Parmar J (2020). Longitudinal Competence Programs for Basic Point-of-Care Ultrasound in Critical Care: A Systematic Review. Chest.

[33] Australasian Society of Ultrasound in Medicine (ASUM). Certificate for Allied Health Performed Ultrasound (CAHPU). The Australasian Society of Ultrasound in Medicine (ASUM). 2020. https://www.asum.com.au/education/cahpu-course/. Accessed 1 May 2021.

[34] Australasian Society of Ultrasound in Medicine (ASUM). Certificate in Clinical Performed Ultrasound (CCPU). The Australasian Society of Ultrasound in Medicine (ASUM). https://www.asum.com.au/education/ccpu-course/. Accessed 1 May 2021.

[35] Doig M, Dizon J, Guerrero K, Parange N (2019). Exploring the availability and impact of antenatal point-of-care ultrasound services in rural and remote communities: A scoping review. Australas J Ultrasound Med.

[36] Westerway SC (2019). Comparing the effectiveness of training course formats for point-of-care ultrasound in the third trimester of pregnancy. Australas J Ultrasound Med.

[37] Battye K, Roufeil L, Edwards M, et al. SARRAH- Strategies for increasing allied health recruitment and retention in rural Australia: A Rapid Review. Services for Australian Rural and Remote Allied Health (SARRAH). 2019. https://sarrah.org.au/images/rapid_review_-_recruitment_and_retention_strategies_-_final_web_ready.pdf. Accessed 19 Jan 2022.

[38] Campos-Zamora M, Gilbert H, Esparza-Perez RI (2022). Continuing professional development challenges in a rural setting: A mixed-methods study. Perspect Med Educ.

[39] Australasian Society of Ultrasound in Medicine (ASUM). A guide to providing an ultrasound workshop. Australasian Society of Ultrasound in Medicine (ASUM). 2016. https://www.google.com/search?client=firefox-b-d&q=A+Guide+to+Providing+an+Ultrasound+Workshop#. Accessed 11 May 2021.

[40] Kumar A, Kugler J, Jensen T (2019). Evaluation of Trainee Competency with Point-of-Care Ultrasonography (POCUS): a Conceptual Framework and Review of Existing Assessments. J Gen Intern Med.

[41] Abuhamad A, Minton KK, Benson CB (2018). Obstetric and Gynecologic Ultrasound Curriculum and Competency Assessment in Residency Training Programs: Consensus Report: Obstetric and Gynecologic Ultrasound Training. J Ultrasound Med.

[42] CAE. Vimedix ultrasound simulator. CAE Healthcare. 2019. https://www.caehealthcare.com/media/files/TechSheets/Vimedix-ObGyn-TechSheet.pdf. Accessed 23 Aug 2021.

[43] Reio TG, Rocco TS, Smith DH, Chang E (2017). A Critique of Kirkpatrick’s Evaluation Model. New Horizons Adult Educ Human Res Dev.

[44] Moreau KA (2017). Has the new Kirkpatrick generation built a better hammer for our evaluation toolbox?. Med Teach.

[45] Jim K, Wendy K. An Introduction to the New World Kirkpatrick Model. Kirkpatrick Partners. 2019. https://www.kirkpatrickpartners.com/Portals/0/Resources/White%20Papers/Introduction%20to%20the%20Kirkpatrick%20New%20World%20Model.pdf. Accessed 16 Aug 2021.

[46] Equator Network. The Strengthening the Reporting of Observational Studies in Epidemiology (STROBE) Statement: guidelines for reporting observational studies. UK EQUATOR Centre. 2021. https://www.equator-network.org/reporting-guidelines/strobe/. Accessed 15 Sept 2021.

[47] Smith A, Parsons M, Renouf T, Boyd S, Rogers P (2019). A mixed-methods evaluation of a multidisciplinary point of care ultrasound program. Med Teach.

[48] Kolbe N, Killu K, Coba V (2015). Point of care ultrasound (POCUS) telemedicine project in rural Nicaragua and its impact on patient management. J Ultrasound.

[49] Shaw-Battista J, Young-Lin N, Bearman S, Dau K, Vargas J (2015). Interprofessional Obstetric Ultrasound Education: Successful Development of Online Learning Modules; Case-Based Seminars; and Skills Labs for Registered and Advanced Practice Nurses, Midwives, Physicians, and Trainees. J Midwifery Womens Health.

[50] Stolz LA, Muruganandan KM, Bisanzo MC (2015). Point-of-care ultrasound education for non-physician clinicians in a resource-limited emergency department. Trop Med Int Health.

[51] Wanjiku GW, Bell G, Wachira B (2018). Assessing a novel point-of-care ultrasound training program for rural healthcare providers in Kenya. BMC Health Serv Res.

[52] Bell G, Wachira B, Denning G (2016). A pilot training program for point-of-care ultrasound in Kenya. Afr J Emerg Med.

[53] Vinayak S, Brownie S (2018). Collaborative task-sharing to enhance the Point-Of-Care Ultrasound (POCUS) access among expectant women in Kenya: The role of midwife sonographers. J Interprof Care.

[54] Dalmacion GV, Reyles RT, Habana AE (2018). Handheld ultrasound to avert maternal and neonatal deaths in 2 regions of the Philippines: an iBuntis intervention study. BMC Pregnancy Childbirth.

[55] Rominger AH, Gomez GAA, Elliott P (2018). The implementation of a longitudinal POCUS curriculum for physicians working at rural outpatient clinics in Chiapas, Mexico. Crit Ultrasound J..

[56] Baltarowich OH, Goldberg BB, Wilkes AN, Anane-Firempong A, Veloski JJ. Effectiveness of “teaching the teachers” initiative for ultrasound training in Africa. Acad Radiol. 2009;16(6):758–62. 10.1016/j.acra.2008.12.023. 10.1016/j.acra.2008.12.023 19362026

[57] Lee JB, Tse C, Keown T (2017). Evaluation of a point of care ultrasound curriculum for Indonesian physicians taught by first-year medical students. World J Emerg Med.

[58] Mandavia DP, Aragona J, Chan L, Chan D, Henderson SO (2000). Ultrasound training for emergency physicians–a prospective study. Acad Emerg Med.

[59] Nathan RO, Swanson JO, Swanson DL (2017). Evaluation of Focused Obstetric Ultrasound Examinations by Health Care Personnel in the Democratic Republic of Congo, Guatemala, Kenya, Pakistan, and Zambia. Curr Probl Diagn Radiol.

[60] Dornhofer K, Farhat A, Guan K (2020). Evaluation of a point-of-care ultrasound curriculum taught by medical students for physicians, nurses, and midwives in rural Indonesia. J Clin ultrasound JCU.

[61] Kotagal M, Quiroga E, Ruffatto BJ (2015). Impact of point-of-care ultrasound training on surgical residents’ confidence. J Surg Educ.

[62] Shah S, Santos N, Kisa R (2020). Efficacy of an ultrasound training program for nurse midwives to assess high-risk conditions at labor triage in rural Uganda. PLoS ONE.

[63] Shah S, Adedipe A, Ruffatto B (2014). BE-SAFE: Bedside sonography for assessment of the fetus in emergencies: educational intervention for late-pregnancy obstetric ultrasound. West J Emerg Med.

[64] Kimberly HH, Murray A, Mennicke M (2010). Focused maternal ultrasound by midwives in rural Zambia. Ultrasound Med Biol.

[65] Burns A, Whittaker J, Tomevska S, et al. Regional hospitals compared with third world as doctors put pressure on NSW Government to call for judicial inquiry. Australian Broadcast Agency (ABC). 2019 updated 1 November 2019. https://www.abc.net.au/news/2019-11-01/doctors-call-for-judicial-inquiry-into-regional-health/11630746. Accessed 9 Apr 2021.

[66] Micks T, Sue K, Rogers P (2016). Barriers to point-of-care ultrasound use in rural emergency departments. CJEM.

[67] Landry A, Eicken J, Dwyer K (2017). Ten Strategies for Optimizing Ultrasound Instruction for Group Learning. Cureus..

[68] Canellas M, Kendall JL (2018). The Flipped Classroom: Addressing the Ultrasound Curriculum Gap in Undergraduate Medical Education. Med Sci Educ.

[69] Vinayak S, Sande J, Nisenbaum H, Nolsoe CP (2017). Training Midwives to Perform Basic Obstetric Point-of-Care Ultrasound in Rural Areas Using a Tablet Platform and Mobile Phone Transmission Technology-A WFUMB COE Project. Ultrasound Med Biol.

[70] Lewiss RE, Hoffmann B, Beaulieu Y, Phelan MB (2014). Point-of-care ultrasound education: the increasing role of simulation and multimedia resources. J Ultrasound Med.

[71] Chalouhi GE, Bernardi V, Ville Y (2015). Ultrasound simulators in obstetrics and gynecology: state of the art. Ultrasound Obstet Gynecol.

[72] Bradley K, Quinton A, Aziz A (2019). Determining if simulation is effective for training in ultrasound: A narrative review. Sonography.

[73] Tolsgaard MG (2018). Assessment and learning of ultrasound skills in Obstetrics & Gynecology. Danish Med J.

[74] Chalouhi GE, Bernardi V, Gueneuc A (2016). Evaluation of trainees’ ability to perform obstetrical ultrasound using simulation: challenges and opportunities. Am J Obstet Gynecol..

[75] The National Rural Health Alliance (NRHA) (2017). Why we need a new rural and remote health strategy June: 2017.

[76] Australian Institute of Health and Welfare (AIHW) (2019). Rural & remote health.

[77] Salimi N, Gonzalez-Fiol A, Yanez D (2022). Ultrasound Image Quality Comparison Between a Handheld Ultrasound Transducer and Mid-Range Ultrasound Machine: Image characteristics of the Butterfly iQ vs. Sonosite M-Turbo. POCUS J.

[78] Rykkje A, Carlsen JF, Nielsen MB (2019). Hand-Held Ultrasound Devices Compared with High-End Ultrasound Systems: A Systematic Review. Diagnostics (Basel, Switzerland)..

[79] Loria K. Remote Access: How Ultrasound in Telemedicine is Changing Education, Training, and Patient Care. Great Valley Publishing Company 2018. https://www.radiologytoday.net/archive/rt0818p24.shtml. Accessed 6 May 2021.

[80] Del Rio M, Meloni V, Frexia F (2018). Augmented reality for supporting real time telementoring: An exploratory study applied to ultrasonography.

[81] Wang S, Parsons M, Stone-McLean J (2017). Augmented Reality as a Telemedicine Platform for Remote Procedural Training. Sensors (Basel).

[82] Engum SA, Jeffries PR (2012). Interdisciplinary collisions: Bringing healthcare professionals together. Collegian.

