# Supplementary material for: Evaluation of antenatal point-of-care ultrasound training workshops for rural/remote healthcare clinicians: a prospective single cohort study
Source: BMC Med Educ. 2022 Dec 30;22:906. doi: 10.1186/s12909-022-03888-5 (PMC9805197; doi:10.1186/s12909-022-03888-5)
Supplement: Supplementary file 1 — Additional file 1: Table 1. General workshop requirements defined by ASUM. [file 12909_2022_3888_MOESM1_ESM.pdf]

Additional Table 1: General workshop requirements defined by *ASUM

ASUM general workshop requirements[39]
Faculty- must include a medical specialist with appropriate and extensive clinical experience/qualifications. Instructors must have significant practical experience in the application being taught. Registered sonographers can assist with teaching skills.
Teaching (including practical) hours should at least meet those published in ASUM credentialing syllabus for the application taught.
Provision of course syllabus, learning materials, recommended texts and other references.
Instructor to trainee ratio 1:5.
Machine to trainee ratio 1:5.
Appropriate models and patients.
Setting to accommodate lectures and practical scanning sessions.
Pre- and post-course tests.
Evidence of attendance including course hours.

**ASUM- Australasian Society of Ultrasound in Medicine*
