# Supplementary material for: Evaluation of antenatal point-of-care ultrasound training workshops for rural/remote healthcare clinicians: a prospective single cohort study
Source: BMC Med Educ. 2022 Dec 30;22:906. doi: 10.1186/s12909-022-03888-5 (PMC9805197; doi:10.1186/s12909-022-03888-5)
Supplement: Supplementary file 2 — Additional file 2: Table 2. Methods for assessing PoCUS competency. [file 12909_2022_3888_MOESM2_ESM.pdf]

Additional Table 2: Methods for assessing Point-of-Care (PoCUS) competency

Methods for assessing competency in PoCUS[40,41]
Technical competency assessment- probe selection, image mode selection (e.g., cardiac, obstetric), proper image orientation, probe positioning, depth, gain, centering of target structure, demonstrates advanced functions (M-mode, Doppler, image capture), troubleshooting.
Knowledge assessment<br>Course entry assessment following pre-reading<br>Pre and post course assessment<br>Multiple-choice questions<br>Written answer
Objective Structured Clinical Examination (OSCE)<br>Practical examination- Formative and summative<br>Oral exam
Standardized checklists for evaluating technical skill
Skill assessment on *simulator, model, or standardised patient
Review of images obtained on real patients (logbook review)
Real-time assessment of scanning actual patients and clinical decision making
Longitudinal patient evaluation and periodic review to assess accuracy of PoCUS interpretation
Self-assessment<br>knowledge/skill<br>perceptions/attitudes<br>Change in work behaviour/scanning frequency

** ISUOG encourages the use of simulation in training and competency assessment in early training[40]*
