# Supplementary material for: Evaluation of antenatal point-of-care ultrasound training workshops for rural/remote healthcare clinicians: a prospective single cohort study
Source: BMC Med Educ. 2022 Dec 30;22:906. doi: 10.1186/s12909-022-03888-5 (PMC9805197; doi:10.1186/s12909-022-03888-5)
Supplement: Supplementary file 3 — Additional file 3. Post-workshop evaluation form. [file 12909_2022_3888_MOESM3_ESM.pdf]

HNP Post Workshop Evaluation

Where did you hear about this workshop?

Ultrasound scanning experience (please circle):

None Less than 6 months 6-12 months 1-2 years 2 years+

INSTRUCTIONS

Please rate aspects of the workshop on a 1 to 5 scale:
SA = "Strongly agree," or the highest, most positive impression
A = “Agree”
N= "Neither agree nor disagree"
D = “Disagree”
SD = "Strongly disagree," or the lowest, most negative impression

Workshop CONTENT (Circle your response to each item)

1. I was well informed of the objectives of this workshop	SA	A	N	D	SD
2. This workshop is relevant to my learning objectives as stated in the course	SA	A	N	D	SD

Workshop DESIGN (Circle your response to each item)

3. The workshop activities stimulated my learning	SA	A	N	D	SD
4. The difficulty level of this workshop was appropriate	SA	A	N	D	SD
5. The pace of this workshop was appropriate	SA	A	N	D	SD

Workshop ACTIVITIES (Circle your response to each item)

6. The morphology VIMEDIX simulator was useful to acquire fetal views	SA	A	N	D	SD
7. The simulators were an advantage to the workshop	SA	A	N	D	SD

Workshop FACULTY (Circle your response to each item)

8. The presenters in this workshop encouraged active audience participation and interaction	SA	A	N	D	SD
9. The presenters devoted enough time for clarification of queries	SA	A	N	D	SD

Workshop IMPACT (Circle your response to each item)

10. It increased my confidence in my abilities to scan	SA	A	N	D	SD
11. I accomplished the objectives of this workshop	SA	A	N	D	SD
12. I will be able to use what I learned in this workshop	SA	A	N	D	SD

HNP Post Workshop Evaluation

HOW WOULD YOU IMPROVE THIS WORKSHOP?

Please respond to the below questions in terms of the following:

- Information provided
- Content covered
- Instructional methods
- Hands-on activities
- Time allocation

13. What is most valuable about this workshop?

14. What is least valuable about this workshop?

15. What would you like to see included in the content next time?

16. Any other comments?

Your feedback is sincerely appreciated. Thank you for your time.
