# Supplementary material for: Evaluation of antenatal point-of-care ultrasound training workshops for rural/remote healthcare clinicians: a prospective single cohort study
Source: BMC Med Educ. 2022 Dec 30;22:906. doi: 10.1186/s12909-022-03888-5 (PMC9805197; doi:10.1186/s12909-022-03888-5)
Supplement: Supplementary file 4 — Additional file 4. 6-month follow-up survey form. [file 12909_2022_3888_MOESM4_ESM.pdf]

Healthy Newborn Project - Six month workshop survey- Final

We rely on your feedback

Welcome and thank you for your participation. This survey is being conducted by The Healthy Newborn Project research team from the Division and School of Health Sciences at the University of South Australia (UniSA), School of Health Sciences **to evaluate the impact of the point-of-care antenatal ultrasound training workshop you attended this year.**

Data collected through this survey will provide robust evidence to inform governments of future planning needs and solutions. Aggregate data will be used to provide the investigators and ultrasound instructors with feedback regarding the quality of the program and the collective benefits to participants, and will inform improvements in delivery of future ultrasound training workshops. Obtaining feedback from professionals in front line rural and remote health care is vital to this process. Let your voice be heard.

The survey is estimated to take **approximately 5-10 minutes** of your time and we greatly appreciate your effort in its completion. Please answer all questions.

Confidentiality statement: Completion of the survey indicates informed consent. Individual responses will remain confidential and will be analysed collectively with other participant responses. The researcher will take every care to remove any identifying material from the responses you provide as early as possible and participants will not be identified in the reporting of the research. No information which could lead to the identification of any individual will be released, unless as required by law. However, the researcher cannot guarantee the confidentiality or anonymity of material transferred by email or the internet. Participation is voluntary and you are free to withdraw from the study at any point while completing the survey. It is not anticipated that there are any risks to participation in this study beyond those encountered during everyday life.

All information collected as part of the study will be retained for five years. The data will be stored securely onsite in electronic format on a password protected UniSA computer server. Data will be held on survey monkey platform until exported. Data will be deleted from the survey monkey immediately following collection and export. The data collected will be non-identifiable and could be used in external publications and presentations.

This study is funded by 'The Hospital Research Foundation'. This research is in line with the National Statement on Ethical Conduct in Human Research (2007) - Updated May 2015 (https://www.nhmrc.gov.au/guidelines-publications/e72). And has been approved by the University of South Australia's Human Research Ethics Committee. If you have any ethical concerns about the project or questions about your rights as a participant, or should you or any third parties wish to lodge a complaint about either the study or the way it is being conducted, please contact the Executive Officer of this Committee – Ms Vicki Allen (tel: +61 8 8302 3118; email: humanethics@unisa.edu.au).

Healthy Newborn Project - Six month workshop survey- Final

* 1. This workshop provided most of the information I need to be able to perform antenatal point of care ultrasound while managing antenatal patients.

- Strongly agree
- Agree
- Undecided
- Disagree
- Strongly disagree

* 2. The information and skills provided in this workshop are relevant to my role.

- Strongly agree
- Agree
- Undecided
- Disagree
- Strongly disagree

* 3. I have noticed positive results in my clinical practice as an outcome of the training.

- Strongly agree
- Agree
- Undecided
- Disagree
- Strongly disagree

* **4. I am expecting positive results from this initiative in the future.**

- Strongly Agree
- Agree
- Undecided
- Disagree
- Strongly disagree

* **5. As a result of the workshop, my confidence in antenatal ultrasound scanning has improved.**

- Strongly agree
- Agree
- Undecided
- Disagree
- Strongly disagree

* **6. As a result of the workshop, I have** (check all that apply):

- [ ] Improved reporting skills (documentation and/or verbal) in antenatal ultrasound scanning
- [ ] Increased personal confidence in antenatal ultrasound scanning
- [ ] Improved management and prioritisation of antenatal patients following ultrasound scanning
- [ ] Observed improved patient compliance with antenatal care advise as a result of ultrasound scanning
- [ ] Other (please specify)

* 7. **As a result of the workshop, I am more confident about my assessment, clinical decision making and reporting in the following** (check all that apply):

- [ ] Fetal lie / fetal position / placental position / terminology / amniotic fluid
- [ ] First trimester ultrasound
- [ ] FM, fetal heart and M Mode
- [ ] Second and third trimester biometry
- [ ] Early pregnancy failure / pregnancy of unknown location / ectopic pregnancies
- [ ] Multiple pregnancies

* 8. **As a result of the workshop, I have improved in the following practical/scanning areas** (check all that apply):

- [ ] Basic scanning techniques
- [ ] Fetal Lie
- [ ] Fetal Heart
- [ ] Placental position
- [ ] Cervix
- [ ] Amniotic fluid-AFI and single pocket
- [ ] Fetal heart beat
- [ ] Ectopic pregnancy
- [ ] Free fluid in the pelvis
- [ ] Fetal demise
- [ ] First trimester measurement
- [ ] Twin pregnancy
- [ ] Other (please specify)

* 9. **As a result of attending the workshop** (check all that apply):

- [ ] I am performing scans I would not have previously attempted
- [ ] I am performing antenatal scans that are being used to assist in patient management / clinical diagnosis
- [ ] Scans I have performed have assisted in earlier clinical diagnosis of patients
- [ ] Scans I have performed have resulted in changes to patient management
- [ ] Other (please specify)

* 10. **What is your current role?**

- [ ] Registered Midwife
- [ ] General Practitioner
- [ ] Other (please specify)

* 11. **How many years of clinical experience do you have?**

* 12. **What ultrasound training and experience have you had prior to attending the workshop** (i.e. years and areas of experience, past training/courses)?

* 13. **How often did you perform antenatal ultrasound in your clinical practice before attending the workshop?**

- ○ Never
- ○ Very rarely
- ○ Occasionally
- ○ Frequently

* 14. **How often do you perform antenatal ultrasound in your clinical practice currently?**

- ○ Never
- ○ Very rarely
- ○ Occasionally
- ○ Frequently

* 15. **What information from this workshop has been most relevant to your role?**

* 16. **Was there any information in this workshop that is NOT relevant to your role? If so, what?**

* 17. **The workshop covered 2 days of presentations interspaced by practical demonstrations and scanning sessions, including live and simulated pregnant models. Please provide some feedback on the format.**

* 18. **Looking back over the workshop's theoretical and practical components, what content did you find most useful** (provide any valuable tips you took away)?

* 19. **Looking back, what would you change about this workshop** (consider what you wish had been covered that was not or what could have been covered in more detail)?

* 20. **In your own words, explain why it was important for you to attend the workshop.**

* 21. **Have you applied what you learnt from the workshop in a real life situation?**

* 22. **Are you seeing any impact on patient outcomes as a result of this training? If so, please describe in detail with examples.**

* 23. **What additional training or support do you need to consolidate your knowledge and skills and what would help you use ultrasounds more in your clinical practice?**

* 24. **Since attending the workshop, what would you say has been the main change to your practice?**

* 25. **What are the main barriers you face accessing/maintaining continual professional development (CPD)?**
