# Supplementary material for: Evaluation of antenatal point-of-care ultrasound training workshops for rural/remote healthcare clinicians: a prospective single cohort study
Source: BMC Med Educ. 2022 Dec 30;22:906. doi: 10.1186/s12909-022-03888-5 (PMC9805197; doi:10.1186/s12909-022-03888-5)
Supplement: Supplementary file 5 — Additional file 5. First-trimester OSCE assessment form. [file 12909_2022_3888_MOESM5_ESM.pdf]

Trainee name: _______________________________ **Assessor's name:** ________________________________

OBSTETRICS FIRST TRIMESTER PRACTICAL ASSESSMENT

Please make note of the students experience in obstetric scanning

……….

A 33-year-old lady presents at 8-weeks' gestation with a request form for a dating ultrasound. As you get her into the room, her husband explains that they are very stressed due to previous 2 early miscarriages.

a) How will you reassure the couple and gain their compliance before starting the scan?

	Yes	No	Comment
Trainee's response is appropriate according to their departmental protocol. Along the lines of 'I will have a good look at the scan and explain once I work out the status' or similar			

a) Show me how you would initially evaluate the pregnancy before taking any images. Tell me what you are looking at as you do this.

	Yes	No	Comment
Full initial evaluation of uterus, adnexa and GS performed?			

b) Now that you have done the scout scan, what features will you document regarding the gestation?

	Yes	No	Comment
Whether intrauterine			
Single/multiple			
G sac			
Yolk sac			
Fetal pole			
Cardiac activity			

c) While you are scanning, the lady tells that she has had some spotting for the past 2 days. In addition to the above-mentioned features, what else should you look for and document with this history?

	Yes	No	Comment
Any signs of sub-chorionic haemorrhage<br>Sonographic appearance and significance (hypoechoic, usual spontaneous resolution, can lead to miscarriage)			

d) What are the criteria for correct CRL measurement at 12 weeks? Freeze an image where you would perform a crown-rump measurement and point to where you would place the callipers.

	Yes	No	Comment
Mid sag section<br>Horizontal orientation (ideal)<br>Good definition of end points of crown and rump<br>Neck in neutral position<br>Fetus occupies atleast 2/3rds of image			
Correct placement of calipers			

e) What is the upper limit of CRL measurement that can be documented after which other parameters need to be used?

	Yes	No	Comment
CRL> or = 84mm			

f) What is the suggested ideal method to document fetal cardiac activity in early 1st trimester? Justify your answer

	Yes	No	Comment
M mode<br>ALARA principle			

Trainee results

**Domain**	**Good**	**Borderline**	**Poor**
Scan technique			
Communication (patient autonomy respected)			
Accurate measurements obtained (correct plane and calliper placement for required structures, demonstrated knowledge of normal limits)			

**Overall performance (circle)**	Satisfactory	Not Satisfactory
