# Supplementary material for: Evaluation of antenatal point-of-care ultrasound training workshops for rural/remote healthcare clinicians: a prospective single cohort study
Source: BMC Med Educ. 2022 Dec 30;22:906. doi: 10.1186/s12909-022-03888-5 (PMC9805197; doi:10.1186/s12909-022-03888-5)
Supplement: Supplementary file 6 — Additional file 6. Second-trimester OSCE assessment form. [file 12909_2022_3888_MOESM6_ESM.pdf]

Trainee name: ______________________________ **Assessor's name:** _______________________________

OBSTETRICS SECOND TRIMESTER PRACTICAL ASSESSMENT

Please make note of the students experience in obstetric scanning-

………

The patient has had a blunt injury and presents at 26 weeks' gestation to assess the status of her fetus.

a) Show me how you would initially evaluate the pregnancy. Tell me what you are looking at as you do this. Tell me how the baby is lying.

	Yes	No	Comment
Full initial evaluation of uterus and fetus performed?			
Correct fetal lie?			

b) Show me where the placenta is, and comment on its location in relation to the cervix. What else would you assess in the lower segment of the uterus?

	Yes	No	Comment
Placenta correctly identified			

c) Name the parameters measured for a standard biometry assessment

	Yes	No	Comment
BPD/ HC/AC/FL			

d) Freeze an image where you would perform BPD and HC measurement. What structures can you see that demonstrate you are in the correct location? Place callipers for BPD

	Yes	No	Comment
Correct location and calliper placement?			
Midline falx, thalami, cavum (no cerebellum)			

e) Freeze an image where you would perform an abdominal circumference measurement. What structures can you see that demonstrate you are in the correct location?

	Yes	No	Comment
Correct location?			
Stomach, spine, intrahepatic portion of the umbilical vein			

f) Demonstrate how you would obtain femur length? Place callipers for FL.

	Yes	No	Comment
Correct image and placement of callipers			

g) Demonstrate how you would check and document fetal heart rate

	Yes	No	Comment
Fetal heart trace OBTAINED on M-mode			

Trainee results

**Domain**	**Good**	**Borderline**	**Poor**
Scan technique			
Communication			
Accurate measurements obtained (correct plane and calliper placement for required structures, demonstrated knowledge of normal limits)			

**Overall performance (circle)**	Satisfactory	Not Satisfactory
