# Supplementary figures and images for: Evaluation of antenatal point-of-care ultrasound training workshops for rural/remote healthcare clinicians: a prospective single cohort study
Source: BMC Med Educ. 2022 Dec 30;22:906. doi: 10.1186/s12909-022-03888-5 (PMC9805197; doi:10.1186/s12909-022-03888-5)

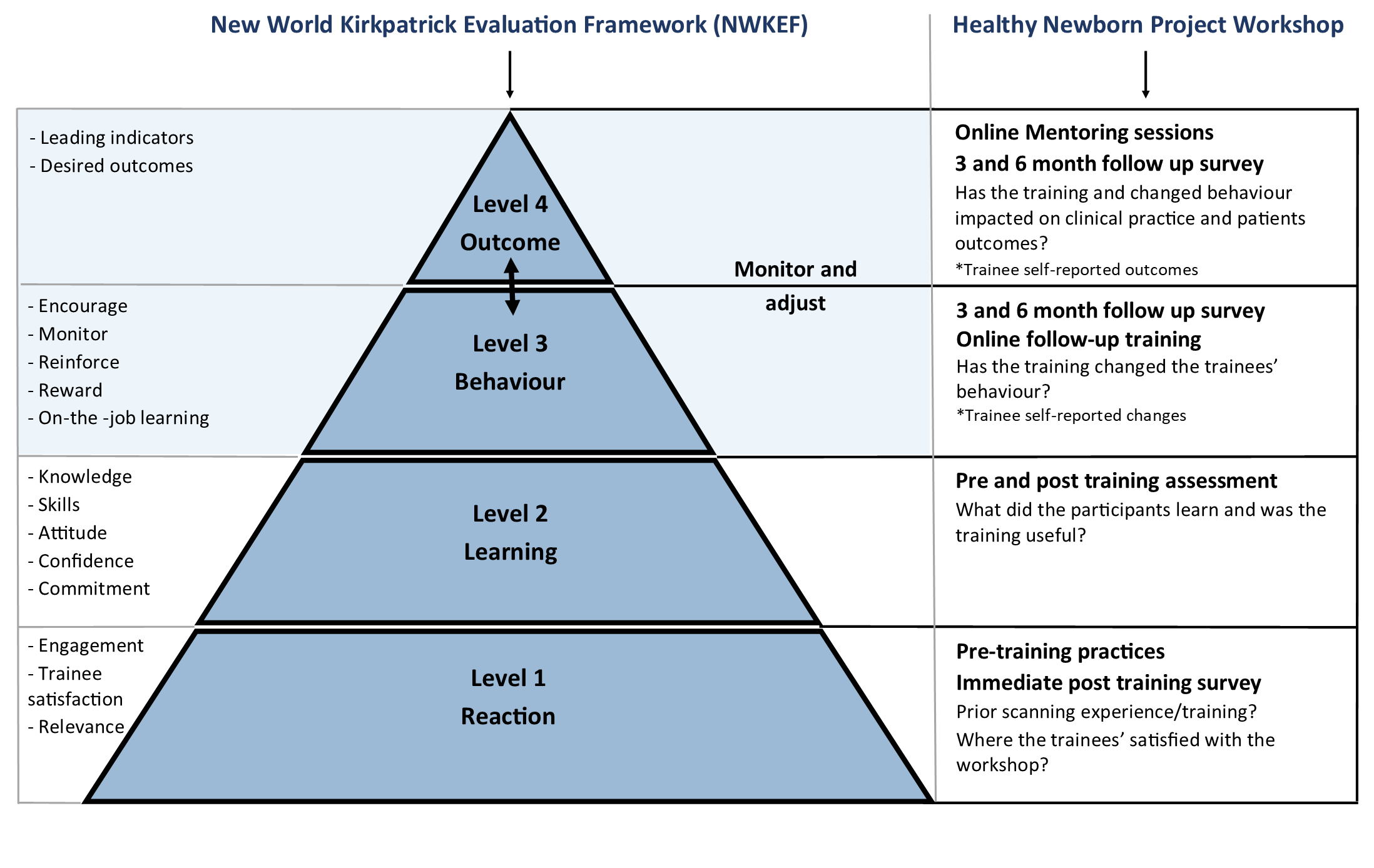

Supplement: Supplementary file 7 — Additional file 7: Figure 1. New World Kirkpatrick Evaluation Framework (NWKEF) [45] for training evaluation and the Healthy Newborn Project workshops. [file 12909_2022_3888_MOESM7_ESM.bmp]
