# Supplementary material for: Evaluation of antenatal point-of-care ultrasound training workshops for rural/remote healthcare clinicians: a prospective single cohort study
Source: BMC Med Educ. 2022 Dec 30;22:906. doi: 10.1186/s12909-022-03888-5 (PMC9805197; doi:10.1186/s12909-022-03888-5)
Supplement: Supplementary file 8 — Additional file 8: Table 3. Trainee demographics (role, clinical experience, clinic remoteness area, pre- and post-workshop test results, previous ultrasound experience and ultrasound unit used). [file 12909_2022_3888_MOESM8_ESM.pdf]

Additional Table 3: Trainee demographics (role, clinical experience, clinic remoteness area, pre- and post-workshop test results, pervious ultrasound experience and ultrasound unit used)

Workshop	Role (GP, M/N)	Clinical experience (Years)	Remoteness area (*ASGS ARIA+ 2016)	Pre-test result %	Post-test result %	Previous PoCUS **training /experience	Ultrasound Unit used clinically
WS1	GP	30	RA3	62.5%	87.5%	Yes	PcAable/Mindray
WS1	GP	22	RA3	62.5%	87.5%	Yes	Sonosite iViz
WS1	GP	40	RA3 RA5	50.0%	87.5%	No	NR
WS1	GP	12	RA3	56.3%	100.0%	Yes	Portable
WS1	GP	22	RA5	62.5%	56.3%	Yes	Sonosite portable, GE Dual Probe VSCAN, Telemed ClarUs EXT-1M
WS1	GP	8	RA3	56.3%	68.8%	Yes	Portable
WS1	M/N	11	RA5	62.5%	68.8%	Yes	Sonosite- Portable
WS1	M/N	12	RA5	43.8%	81.2%	Yes	NR
WS1	M/N	5	RA4	43.8%	75.0%	No	Sonosite- Portable
WS1	M/N	10	RA4 RA5	75.0%	93.8%	Yes	Sonosite M Turbo
WS1	M/N	5	RA4 RA5	62.5%	75.0%	No	Sonosite Edge
WS1	M/N	13	RA5	68.8%	81.2%	Yes	Sonosite M Turbo
WS2	GP	6	RA3	68.8%	93.8%	Yes	Sonosite Edge
WS2	GP	2	RA3	68.8%	81.2%	Yes	Sonosite MicroMaxx
WS2	GP	14	RA3 RA4 RA5	56.3%	87.5%	Yes	Sonosite Edge
WS2	GP	3	RA3 RA4 RA5	50.0%	100.0%	No	Portable
WS2	M/N	5	RA3	50.0%	68.8%	Yes	Sonosite Edge II
WS2	M/N	9	RA4	50.0%	68.8%	Yes	NR
WS2	M/N	13	RA5	37.5%	75.0%	No	Sonosite M Turbo
WS2	M/N	40	RA5	43.8%	43.8%	Yes	Sonosite- Portable
WS2	M/N	5	RA4 RA5	25.0%	93.8%	No	Sonosite- Portable
WS2	M/N	26	RA4 RA5	62.5%	62.5%	Yes	Sonosite- Portable
WS2	M/N	13	RA3 RA4	31.3%	93.8%	No	NR

WS2	M/N	3	RA5	43.8%	81.2%	No	Sonosite M Turbo
WS2	M/N	7	RA5	62.5%	93.8%	No	Sonosite M Turbo
WS3	GP	26	RA5	62.5%	81.2%	Yes	Sonosite M Turbo
WS3	GP	11	RA5	75.0%	81.2%	Yes	Sonosite iViz
WS3	GP	7	RA5	81.2%	75.0%	No	Sonosite Edge II
WS3	GP	37	RA5	62.5%	62.5%	Yes	Sonosite Edge
WS3	GP	10	RA5	62.5%	93.8%	Yes	Sonosite Edge
WS3	GP	45	RA3	62.5%	87.5%	Yes	Sonosite Turbo Edge I
WS3	M/N	9	RA5	62.5%	75.0%	Yes	Sonosite X-Porte
WS3	M/N	16	RA4	56.3%	62.5%	Yes	Portable
WS3	M/N	13	RA5	37.5%	68.8%	Yes	Sonosite
WS3	M/N	10	RA5	31.3%	62.5%	Yes	Sonosite X-Porte
WS3	M/N	28	RA5	56.3%	75.0%	Yes	Sonosite X-Porte
WS3	M/N	17	RA4	50.0%	62.5%	No	Sonosite X-Porte
WS3	M/N	10	RA5	25.0%	56.3%	No	Sonosite – M-Turbo
WS3	M/N	40	RA5	56.3%	68.8%	No	Portable
WS3	M/N	8	RA5	50.0%	81.2%	Yes	Sonosite Edge
WS3	M/N	10	RA5	50.0%	56.3%	No	GE Healthcare Venue 40
		Mean 15.4 years		Mean (SD) 54.6% (13.18)	Mean (SD) 77.0% (13.50)	14/41 (34.0%) Inexperienced	

*The Australian Statistical Geography Standard's (ASGS) Accessibility Remoteness Index of Australia (ARIA+) defines 5 geographical categories or remoteness areas (RAs) determined by road distance from the closest urban centre.

** training experience- includes any previous formal ultrasound training course/s including non-obstetric, and clinical scanning experience (i.e. on the job ultrasound use, instruction by colleague).

NR = Not reported

Attended follow-up workshop

GP- General Practitioner

M/N- Midwife/Nurse
