# Supplementary material for: Evaluation of antenatal point-of-care ultrasound training workshops for rural/remote healthcare clinicians: a prospective single cohort study
Source: BMC Med Educ. 2022 Dec 30;22:906. doi: 10.1186/s12909-022-03888-5 (PMC9805197; doi:10.1186/s12909-022-03888-5)
Supplement: Supplementary file 9 — Additional file 9: Table 4. Pre-post test scores for all workshops, and initial workshops by position/role and previous ultrasound experience. [file 12909_2022_3888_MOESM9_ESM.pdf]

Additional Table 4: Pre-post test scores for all workshops, and initial workshops by position/role and previous ultrasound experience

	Workshops 1-3 - Initial training				
	**All trainees (n=41)**	**GP (n=16)**	**M/N (n=25)**	**Prior training /experience (n=27)**	**Inexperienced (n=14)**
**Mean % Pre-course test score (SD)**	54.6 (13.2)	62.5 (8.2)	49.5 (13.37)	58.1 (10.5)	47.8 (15.4)
**Mean % Post-course test score (SD)**	77.0 (13.5)	83.2 (12.4)	73.0 (12.8)	76.4 (13.4)	78.1 (14.2)
**Diff (95% CI)**	22.4 (17.1-27.8)	20.7 (11.8-29.6)	23.5 (16.3-30.6)	18.3 (13.2-23.3)	30.4 (18.0-42.7)
**p-value**	0.00000000018	0.00018	0.00000052	0.000000064	0.00014

	Workshop 4 -Follow-up training group			Initial workshop results for follow-up group		
	**Combined (n=9)**	**GP (n=1)**	**M/N (n=8)**	**Combined (n=9)**	**GP (n=1)**	**M/N (n=8)**
**Mean % Pre-course test score (SD)**	80.6 (12.2)	100	78.1 (10.6)	55.6 (16.1)	68.8	53.9 (16.4)
**Mean % Post-course test score (SD)**	89.6 (8.3)	100	88.3 (7.8)	79.2 (17.7)	93.8	77.3 (18.0)
**Diff (95% CI)**	9.03 (3.1-15.0)	0	10.2 (4.0-16.46)	23.6 (7.4-39.9)	25	23.4 (4.6-42.3)
**p-value**	0.0080		0.0061	0.010		0.022

GP- General Practitioners; M/N- Midwife/Nurse
