# Supplementary material for: Evaluation of antenatal point-of-care ultrasound training workshops for rural/remote healthcare clinicians: a prospective single cohort study
Source: BMC Med Educ. 2022 Dec 30;22:906. doi: 10.1186/s12909-022-03888-5 (PMC9805197; doi:10.1186/s12909-022-03888-5)
Supplement: Supplementary file 10 — Additional file 10: Table 5. Areas of improved confidence and practical scanning. [file 12909_2022_3888_MOESM10_ESM.pdf]

Additional Table 5: Areas of improved confidence and practical scanning

CONFIDENCE		PRACTICAL SCANNING	
As a result of the workshop, I am more confident in assessment, clinical decision making and reporting in the following areas-	*Response (%)*	As a result of the workshop, I have improved in the following practical/scanning areas-	*Response (%)*
Fetal lie / fetal position / placental position / terminology / amniotic fluid	82.0% (32/39)	Basic scanning techniques	97.4% (39/39)
First trimester ultrasound	69.2% (27/39)	Fetal heart / M mode	76.9% (30/39)
FM, fetal heart and M Mode	59.0% (23/39	Fetal lie	75.4% (29/39)
Multiple pregnancies	41.0% (16/39)	Placental position	66.7% (26/39)
Early pregnancy failure / pregnancy of unknown location / ectopic pregnancies	35.9% (14/39)	Cervix	48.7% (19/39)
Second and third trimester biometry	33.3% (13/39)	First trimester measurement	43.6% (17/39)
		Amniotic fluid-AFI and single pocket	38.5% (15/39)
		Twin pregnancy	25.6% (10/39)
		Ectopic pregnancy / Fetal demise	23.1% (9/39)
		Free fluid in the pelvis	15.4% (6/39)

Self-reported confidence and scanning improvement- Responses from 6-month survey- n=39
