# Supplementary material for: Evaluation of antenatal point-of-care ultrasound training workshops for rural/remote healthcare clinicians: a prospective single cohort study
Source: BMC Med Educ. 2022 Dec 30;22:906. doi: 10.1186/s12909-022-03888-5 (PMC9805197; doi:10.1186/s12909-022-03888-5)
Supplement: Supplementary file 11 — Additional file 11: Table 6a. Application of learning/PoCUS to clinical practice: Qualitative responses from 3- and 6-month follow-up surveys. Table 6b. Impact of training/PoCUS use on patient outcomes: Qualitative responses from 3- and 6-month follow-up surveys. [file 12909_2022_3888_MOESM11_ESM.pdf]

Sample of qualitative responses from 3 and 6 month follow-up surveys

Additional Table 6a: Application of learning/PoCUS to clinical practice

Have you applied what you learnt from the workshop in a real life situation?				
**6 month survey**				
**Workshop attended**	**Role**		**If yes - Please describe how you have used what you learnt in your clinical practice.<br>If no - Please specify why you have not applied what you learnt to your clinical practice.**	**Themes**<br>**Improvements**<br>**Applications**<br>**Barriers**
WS1	RM	Y	Yes, many times. Have had more confidence to repeat first trimester dating if not visible, have been confident that I'm looking in the uterus but no sac present, been confident to order additional pathology if concerned (not delay). E.g. better access to appropriate referrals in a timely manner, better estimation of due date (correlates with NT scan if done).	▪ Increased confidence<br>▪ Timely referral<br>▪ Improved estimated due dates<br>▪ Estimated gestation/due dates
WS1	GP	Y	I am applying my skills in clinical practice and gaining confidence in the process. E.g. Encourages patient engagement and reduces the need to travel for ultrasound assessment. Therefore improves monitoring.	▪ Increased confidence<br>▪ Increased patient satisfaction & engagement<br>▪ Increased patient satisfaction & engagement<br>▪ Reduced patient travel<br>▪ Improved monitoring
WS1	RM	N	It has been difficult to apply to practice for dating ultrasounds, due to lack of practice and experience. It would be useful to have someone trained in ultrasound assisting me while attending scans. Sometimes my first contact with women they have already had a dating scan or first trimester screening. Most women who we see need to go to town for their 1st trimester screening or morphology.	▪ Opportunity to scan/practice<br>▪ Need for onsite supervision
WS1	GP	Y	I have been able to utilise the US skills in quite a few ladies - but especially a lady with previous foetal loss providing extra US scans to reassure and check baby. Also managed locally a lady with foetal demise at 30 weeks with initial US by me - and increased confidence with my findings, even prior to confirmation with formal US.	▪ Increased confidence<br>▪ Reassurance/Reduced patient anxiety<br>▪ Fetal viability<br>▪ Fetal demise
WS1	RM	Y	I have learned techniques on how to get the fetus into optimal positions on the screen to be able to measure properly. E.g. Yes, my accuracy in dating USS has improved as well as measurements.	▪ Increased accuracy<br>▪ Increased confidence<br>▪ Improved estimated due dates<br>▪ Estimated gestation/due dates<br>▪ Fetal biometry
WS1	RM	Y	All dating scans are now done in community and only high risk go to referral hospitals or twins, no foetal pole. E.g. better antenatal care with earlier dating scans also women who want a termination now have more options by my scanning earlier.	▪ Improved ANC/planning<br>▪ Reduced patient travel<br>▪ Improved estimated due dates<br>▪ Timely referral<br>▪ Estimated gestation/due dates
WS1	RM	Y	Checking fetal lie with suspected breech presentation, confirmed on ultrasound. Confirming fetal viability.	▪ Timely referral

				▪ Improved ANC/planning<br>▪ Fetal viability<br>▪ Fetal lie
WS1	GP	Y	I scanned a 3rd trimester pregnancy - no issues aside from high BMI. My ultrasound is limited by its poor quality.	▪ Equipment
WS2	RM	Y	I have completed many first and second trimester scans and it has helped commencement of pregnancy care. E.g. women not only get to see their baby and engage with their pregnancy in a whole new way but by seeing where a woman is in her pregnancy means I can more easily prioritise pregnancy care.	▪ Improved ANC/planning<br>▪ Increased patient satisfaction & engagement
WS2	RM	Y	Attempt scanning, even if for very short time, to increase confidence. E.g. better communication/interest from the mother in their antenatal care.	▪ Increased confidence<br>▪ Increased patient satisfaction & engagement
WS2	RM	Y	Determining fetal lie prior to elective caesarean section. E.g. Flagging concerns with Doctors, planning care and change of management, reassuring women.	▪ Improved ANC/planning<br>▪ Timely referral<br>▪ Reassurance/Reduced patient anxiety<br>▪ Fetal lie
WS2	GP	Y	Growth scans when symphysial fundal height seems abnormal. E.g. able to reassure patients.	▪ Reassurance/Reduced patient anxiety<br>▪ Improved monitoring<br>▪ Fetal growth
WS2	GP	Y	Amniotic fluid checks in possible preterm rupture of membranes, confirming fetal lie and fetal heartbeat. E.g. more confidence in dealing with potential pre-term labours.	▪ Increased confidence<br>▪ Amniotic fluid assessment<br>▪ Fetal lie<br>▪ Fetal heart
WS2	RM	Y	Early US. E.g. I am able to assess the gestation of the fetus and plan appropriate and timely antenatal care.	▪ Improved estimated due dates<br>▪ Improved ANC/planning<br>▪ Timely referral<br>▪ Estimated gestation/due dates
WS2	RM	Y	I have performed dating scans on some women, preventing them from needing (potentially) additional trips to town…. I have also used US to check presentation of fetus' when unsure of presentation later on in the pregnancy, preventing a trip to town for a formal US. E.g. improved timeliness of interventions and reassurance as appropriate. E.g. patients are happier to come to clinic.	▪ Improved estimated due dates<br>▪ Reduced patient travel<br>▪ Increased patient satisfaction & engagement<br>▪ Improved ANC compliance/attendance<br>▪ Improved ANC/planning<br>▪ Timely referral<br>▪ Estimated gestation/due dates<br>▪ Fetal lie
WS2	RM	Y	Prior to performing an induction of labour (IOL) on a patient, my abdominal palp felt like a breech presentation, US confirmed breech presentation and a lower segment caesarean section was performed the following day and the IOL was abandoned. I almost had an opportunity to confirm or deny an ectopic pregnancy, however the patient declined the scan. E.g. A small impact, especially when I am able to use the US during antenatal clinic. Discussing fetal position at term and suggesting techniques to get babe into optimal fetal position.	▪ Patient education<br>▪ Timely referral<br>▪ Improved ANC/planning<br>▪ Fetal lie
WS2	RM	Y	Determining fetal lie, placental position, placenta praevia, attempts at checking AFI.	▪ Fetal lie<br>▪ Placental position

				▪ Placenta praevia<br>▪ Amniotic fluid assessment
WS2	GP	N	Limited physical contact with antenates.	▪ Opportunity to scan/practice
WS2	RM	Y	Shown newly pregnant mums a scan. E.g. Better engagement/interest in their pregnancy	▪ Increased patient satisfaction & engagement
WS3	RM	Y	Absolutely, each antenatal appointment I assess the likelihood of whether an ultrasound scan would aid in the care offered or decisions required to provide optimal care to the woman and her baby.	▪ Improved ANC/planning<br>▪ Timely referral
WS4	GP	Y	I'm able to further assess abnormal symphysial fundal height and perform scan to prioritise further assessment/management.	▪ Improved ANC/planning<br>▪ Timely referral<br>▪ Fetal growth
WS4	RM	Y	Yes, I have diagnosed both an ectopic and Molar pregnancy and referred to O & G straight away.	▪ Timely referral<br>▪ Ectopic/molar pregnancy
**3 month survey**				
WS1	RM	Y	I was able to see a fetal demise due to decreased echogenicity and shape of gestational sac. I was confident in my assessment and therefore took further pathology tests and arranged for referral for this woman. I was able to compare the sac with another scan I had performed the same day and I could clearly see the difference between the two scans- previously I feel like I would have doubted my skills and repeated the scan in a week or two.	▪ Increased confidence<br>▪ Improved ANC/planning<br>▪ Timely referral<br>▪ Fetal demise
WS1	GP	Y	Ability to prevent patients travelling as able to confirm fetal viability	▪ Reduced patient travel<br>▪ Fetal viability
WS1	GP	N	No opportunity as yet.	▪ Opportunity to scan/practice
WS1	GP	Y	I had a 31 week pregnancy with no Fetal movement for 3 days. I was able to use the US to confidently check for FH/movements so quick diagnosis and management was possible.	▪ Reassurance/Reduced patient anxiety<br>▪ Improved ANC/planning<br>▪ Timely referral<br>▪ Fetal viability
WS1	RM	Y	I have diagnosed a missed miscarriage, this allowed me to provide timely follow up for the woman and get her to hospital and be seen straight away.	▪ Timely referral<br>▪ Miscarriage
WS1	RM	N	Ultrasound machine no longer functional	▪ Equipment
WS1	GP	N	My ultrasound is poor.	▪ Equipment
WS2	RM	Y	I have only done one early USS since the course as I have only been in my remote role one week since the course. I have attended one early USS. I was able to see the fetus and get a CRL however I found it difficult as it was 3 months since the course and the fetus was very active!! I have only been back in the remote one week since the course so have not performed as much antenatal US as I would have liked so is difficult to answer.	▪ Opportunity to scan/practice
WS2	RM	Y	I feel more comfortable with positioning and basic use of the probe. I feel not as comfortable with the machine as I wished to be therefore do not attempt to take measurements as such. Also because women are often in a hurry due to transport issues.	▪ Increased confidence<br>▪ Equipment<br>▪ Time limits during consultation
WS2	RM	Y	I have been using the machine in my antenatal clinic to improve and keep this confidence up	▪ Increased confidence

WS2	GP	Y	18/40 small Antepartum haemorrhage - my ultrasound demonstrated placenta previa - confirmed on formal ultrasound.	▪ Timely referral<br>▪ Improved ANC/planning<br>▪ Placenta previa
WS2	GP	Y	I am measuring Amniotic Fluid Index/Deepest pocket quite frequently now in our attendees with query preterm labour.	▪ Timely referral<br>▪ Improved ANC/planning<br>▪ Amniotic fluid Index assessment
WS2	RM	Y	I perform dating scans at initial consultations, and point of care scans when women have had concerns such as bleeds to help determine the immediate course of action in accordance to current policy and guidelines. It took a while to acquire an US machine. I have now borrowed one from a different region.	▪ Improved ANC/planning<br>▪ Timely referral<br>▪ Reassurance/Reduced patient anxiety<br>▪ Improved estimated due dates<br>▪ Estimated gestation/due dates<br>▪ Equipment
WS2	GP	N	I haven't - and as such, the issue with this is the lack of clarity from my organisation about the confidence in scanning in the beside context, along with relatively small numbers of women at the moment. While I think the technical skills imparted were of an extremely high standard, with excellent theory on protocol and the parts of assessment, the main barrier at the moment is our institutional assessment of liability and risk.	▪ Legal/liability concerns<br>▪ Employer/management support
WS2	RM	Y	I have performed a few early dating scans, in order to obtain and estimated due date. This information has helped to plan dates for nuchal translucency scans.	▪ Improved ANC/planning<br>▪ Improved estimated due dates<br>▪ Estimated gestation/due dates
WS2	RM	Y	I have only had 2 opportunities to scan since the workshop. Diagnosing a breech prior to induction of labour. Scanning transverse babe and advising mother about spinning babies info to change lie of babe prior to labour.	▪ Patient education<br>▪ Fetal lie
WS2	RM	Y	Checking placenta positioning, when the sonographer wasn't available, checking fetal lie a couple of times, and AFI checks.	▪ Placental position<br>▪ Fetal lie<br>▪ Amniotic Fluid Index assessment
WS2	RM	Y	Early dating mainly.	▪ Improved estimated due dates<br>▪ Estimated gestation/due dates
WS2	GP	N	Limited patient contact currently.	▪ Opportunity to scan/practice
WS3	RN	N	No, because the machine that we have is different from the one that we used during the workshop.	▪ Opportunity to scan/practice<br>▪ Equipment
WS3	RM	Y	One client was seeking termination after earlier attempts to facilitate when she was in her community were declined, the client arrived at our clinic asking for help with her 3 small children. Many complex physical and psychological risks faced this client who was currently in a domestic violent relationship, another baby was not something she felt she could provide for at this time. On further assessment I could confirm her late gestation of 24+ weeks and explain the law and process if she wanted to continue to seek a termination. This information and her being able to see the baby allowed her to face the situation and consider her options. It is an ongoing situation at this stage she is requiring support +++ and there is limited family commitment or options for kinship care of this baby. The identification of accurate gestation is paramount when women are considering termination, and termination methods. Some barriers I have are regular practice, I am waiting to engage the Radiology dept to ask for more intensive practice with them.	▪ Improved ANC/planning<br>▪ Timely referral<br>▪ Improved estimated due dates<br>▪ Patient education<br>▪ Estimated gestation/due dates<br>▪ Opportunity to scan/practice<br>▪ Socioeconomic

WS3	RM	Y	Diagnosing reduced liquor and another example of a possible Breech	▪ Fetal lie<br>▪ Amniotic fluid Index assessment
WS3	GP	N	No - have not seen an antenatal patient since the workshop. There are currently 3 early pregnancies in my clinic but I haven't been able to review them.	▪ Opportunity to scan/practice
WS3	RM	Y	Obese woman with T2DM, had difficulty palpating abdomen for position and lie and finding Fetal heart rate (FHR) on doppler. I was able to use the ultrasound to reassure both the woman and myself of position of babe and FHR.	▪ Reassurance/Reduced patient anxiety<br>▪ Fetal heart<br>▪ Fetal viability<br>▪ Fetal lie
WS3	RM	Y	Recent spontaneous abortion, couldn't identify foetus for gestational age, no placenta -Formal ultrasound confirmed findings.	▪ Timely referral<br>▪ Miscarriage
WS3	GP	Y	Bleeding in early pregnancy in a known intrauterine pregnancy, I was able to detect a foetal heart, which was reassuring.<br>Client attended third trimester, unknown gestation / placental lie - I attempted third trimester scan, though do to correct probe not being able to be located this was of limited value (we have since found it).	▪ Reassurance/Reduced patient anxiety<br>▪ Fetal viability<br>▪ Equipment<br>▪ Opportunity to scan/practice
WS3	GP	Y	Only partially- I have been in the role of acting medical director so have not done much clinical work.	▪ Opportunity to scan/practice
WS3	GP	Y	Mostly first trimester dating.	▪ Improved estimated due dates<br>▪ Estimated gestation/due dates
WS3	GP	Y	I have measured and documented multiple pregnancies, including a low placenta and a breech, getting better with M-mode FH, practicing biometry (need more practice but getting there).	▪ Increased confidence<br>▪ Placental position<br>▪ Fetal lie<br>▪ Fetal heart
WS4	RM	Y	Determining positioning of fetus, and fluid levels	▪ Fetal lie<br>▪ Amniotic Fluid Index assessment
WS4	RM	Y	Try to when time permits	▪ Time limits during consultation
WS4	RM	Y	Yes but still lack on-site/local mentoring/review of images	▪ Need for onsite supervision

Additional Table 6b: Impact of training/PoCUS use on patient outcomes

Are you seeing any impact on patient outcomes as a result of the training?				
6 month survey				
Workshop attended	Role	Please describe in detail with examples.		Themes<br>Improvements<br>Barriers<br>Applications
WS1	RM	Y	Improved accuracy of dating has led to improved timing for morphology and NT scans in our practice. Better access to appropriate referrals in a timely manner. Having ultrasound in remote clinics is a little incentive for women to attend when the midwife is there - this brings in a population of women who might be a bit lackadaisical with attendance especially in the first trimester.	▪ Improved ANC/planning<br>▪ Timely referral<br>▪ Improved estimated due dates<br>▪ Increased patient satisfaction & engagement<br>▪ Improved ANC compliance/attendance<br>▪ Estimated gestation/due dates
WS1	GP	Y	Encourages patient engagement and reduces the need to travel for ultrasound assessment. Therefore improves monitoring.	▪ Increased patient satisfaction & engagement<br>▪ Reduced patient travel<br>▪ Improved ANC/planning<br>▪ Timely referral<br>▪ Improved monitoring
WS1	RM	N	Not at this stage- as I need further confidence and training in ultrasound skills.	▪ Lack of confidence
WS1	GP	Y	Improved timeliness of interventions and reassurance as appropriate	▪ Improved ANC/planning<br>▪ Timely referral<br>▪ Reassurance/Reduced patient anxiety
WS1	RM	Y	My accuracy in dating USS has improved as well as measurements	▪ Increased accuracy<br>▪ Increased confidence<br>▪ Estimated gestation/due dates<br>▪ Fetal biometry
WS1	RM	Y	Better antenatal care with earlier dating scans also women who want a termination now have more options by my scanning earlier.	▪ Improved ANC/planning<br>▪ Timely referral<br>▪ Improved estimated due dates<br>▪ Estimated gestation/due dates
WS1	RM	N	No ultrasound machine.	▪ Equipment
WS1	GP	N	My ultrasound is poor quality and no emergencies have presented as yet.	▪ Opportunity to scan/practice<br>▪ Equipment
WS2	RM	Y	Women not only get to see their baby and engage with their pregnancy in a whole new way but by seeing where a woman is in her pregnancy means I can more easily prioritise pregnancy care.	▪ Improved ANC/planning<br>▪ Timely referral

				▪ Increased patient satisfaction & engagement
WS2	RM	Y	Better communication/interest from the mother in their antenatal care.	▪ Increased patient satisfaction & engagement
WS2	GP	Y	Able to reassure patients.	▪ Reassurance/Reduced patient anxiety
WS2	GP	Y	I have more confidence in dealing with potential pre-term labours.	▪ Increased confidence
WS2	RM	Y	I am able to assess the gestation of the fetus and plan appropriate and timely AN care	▪ Improved estimated due dates<br>▪ Improved ANC/planning<br>▪ Timely referral<br>▪ Estimated gestation/due dates
WS2	RM	Y	Patients are happier to come to clinic	▪ Increased patient satisfaction & engagement<br>▪ Improved ANC compliance/attendance
WS2	RM	Y	A small impact, especially when I am able to use the US during antenatal clinic. Discussing fetal position at term and suggesting techniques to get babe into optimal fetal position	▪ Patient education<br>▪ Fetal lie
WS2	RM	Y	Flagging concerns with Doctors, planning care and change of management, reassuring women.	▪ Improved ANC/planning<br>▪ Timely referral<br>▪ Reassurance/Reduced patient anxiety
WS2	RM	Y	It's hard to tell as numbers of pregnant women were low for the first half of this year, but recently tripled so I think I will use it even more in the next year.	▪ Opportunity to scan/practice
WS2	GP	N	Unable to apply skills in current role	▪ Opportunity to scan/practice
WS2	RM	Y	Better engagement/interest in their pregnancy	▪ Increased patient satisfaction & engagement
WS3	RN	Y	The clients are more eager to have ante natal checks done	▪ Increased patient satisfaction & engagement<br>▪ Improved ANC compliance/attendance
WS3	RM	Y	The women really engage and love to view the fetus and it improves antenatal attendance. Reassuring to see cephalic position in late pregnancy.	▪ Increased patient satisfaction & engagement<br>▪ Improved ANC compliance/attendance<br>▪ Improved ANC/planning<br>▪ Timely referral<br>▪ Fetal lie
WS3	RM	Y	Better dating USS therefore more accurate EDD	▪ Improved estimated due dates<br>▪ Estimated gestation/due dates
WS3	RM	Y	Yes, all positive impacts. Recently a client presented to our clinic with no previous information on her pregnancy and whilst happy to say hello and freely approached did not have the health literacy to recall her current pregnancy information. With the assistance of the USS information I managed to reassure her of her babies wellbeing and plan ongoing care if she was to stay in town with access to transport and liaison services. The pregnancy was advanced 18-20wks so formal morphology was arranged. This woman did ask that I didn't contact	▪ Reassurance/Reduced patient anxiety<br>▪ Improved estimated due dates<br>▪ Improved ANC/planning<br>▪ Timely referral<br>▪ Estimated gestation/due dates

			the community she resided in due to family working in the clinic and concerns regarding confidentiality as she was yet to disclose her pregnancy broadly.	
WS3	RM	Y	On a few occasions I have been able to provide early dating ultrasounds to establish an EDD for women seeking a termination of pregnancy. Without having the point of care USS available to use these women would have received delays in their care at the nearest regional centre and potentially have had to continue with an unplanned pregnancy. Being able to provide an USS at a woman's first visit is hugely helpful in planning and coordinating care, especially when they have chronic diseases such as T2DM.<br>There are many reasons why women cannot access their regional centre for formal USS's for example, one of my past clients presented for the first time at 20+/40, due to road closures due to weather (impassable), road conditions and men's ceremony (women cannot leave community for weeks) this antenate did not have her first formal visit in the regional centre until 32 weeks. Being able to provide a basic USS on community at least helped to plan timely care.	▪ Improved ANC/planning<br>▪ Timely referral<br>▪ Reassurance/Reduced patient anxiety<br>▪ Improved estimated due dates<br>▪ Estimated gestation/due dates<br>▪ Socioeconomic<br>▪ Cultural<br>▪ Environmental
WS3	RM	Y	I have been able to identify twin fetal heart activity in first trimester confirming pregnancy along with physical assessment in second trimester attending initial ultrasound in advanced pregnancy in third trimester and discussing importance of more frequent visits in final trimester I have had obese diabetic pregnant woman with a difficult abdominal palpating in remote community and was able to identify lie and position of fetus	▪ Patient education<br>▪ Multiple pregnancy<br>▪ Fetal lie
WS3	GP	Y	Definitely enables me to assess pregnant women in a way that was not previously possible and make more informed decisions about transfers. Recently had a woman for whom it was not possible to find a foetal heart with the doppler USS. We were able to use the POC uss and were rapidly able to detect a foetal heart and live active foetus that saved a transfer, hospital resources and maternal anxiety. Also able to reassure a client with severe hyperemesis.	▪ Improved ANC/planning<br>▪ Timely referral<br>▪ Reassurance/Reduced patient anxiety<br>▪ Improved monitoring<br>▪ Reduced patient travel<br>▪ Economic benefit<br>▪ Fetal lie<br>▪ Fetal heart
WS3	GP	Y	Yes; demonstrated foetal movements and normal FHR when the woman was complaining that she had not felt kicking - so preventing a long trip to major centre for the same finding.	▪ Reduced patient travel<br>▪ Economic benefit<br>▪ Reassurance/Reduced patient anxiety<br>▪ Fetal heart<br>▪ Fetal viability
WS4	RM	Y	When working remotely I was able to diagnose pregnancy and get a EDD and plan pregnancy care accordingly.	▪ Improved ANC/planning<br>▪ Timely referral<br>▪ Improved estimated due dates<br>▪ Estimated gestation/due dates
WS4	GP	Y	More appropriate transfers to tertiary centres.	▪ Improved ANC/planning<br>▪ Timely referral<br>▪ Economic benefit<br>▪ Reduced patient travel

WS4	RM	Y	I am, as my skills are becoming better I am able to accurately date pregnancies so that subsequent USS i.e. Nuchal can be booked at the right time saving multiple trips to town, also diagnosing abnormalities in early pregnancy and referring quickly potentially can save lives i.e. if ectopic ruptured.	▪ Increased confidence<br>▪ Improved ANC/planning<br>▪ Timely referral<br>▪ Improved estimated due dates<br>▪ Economic benefit<br>▪ Reduced patient travel<br>▪ Estimated gestation/due dates<br>▪ Ectopic pregnancy
WS4	RM	Y	Women are more keen to engage with antenatal care.	▪ Increased patient satisfaction & engagement<br>▪ Improved ANC compliance/attendance
WS4	RM	Y	Many examples of basic dating scans done, providing accurate dates for women, therefore planning nuchal translucency scans is made much easier. I have since moved roles and am based in town now. But have still used ultrasound for this purpose.	▪ Improved estimated due dates<br>▪ Improved ANC/planning<br>▪ Timely referral<br>▪ Estimated gestation/due dates
WS4	RM	Y	We've had several people have to go for formal early scan with either fetal demise, or variation from normal including one ectopic.	▪ Improved ANC/planning<br>▪ Timely referral<br>▪ Fetal demise<br>▪ Ectopic pregnancy
WS4	RM	Y	Definitely, with increased patient attendance and positive pregnancy outcomes from early dating and interventions.	▪ Increased patient satisfaction & engagement<br>▪ Improved ANC compliance/attendance<br>▪ Improved ANC/planning<br>▪ Timely referral
**3 month survey**				
WS1	GP	Y	Yes - ability to regularly US for one patient in particular who has high levels of anxiety during her pregnancy.	▪ Reassurance/Reduced patient anxiety
WS1	GP	Y	Hospital avoidance. Patients' confidence in myself.	▪ Reassurance/Reduced patient anxiety<br>▪ Reduced patient travel<br>▪ Economic benefit
WS1	RM	Y	Yes I am, I have had a couple of very early pregnancies and the skills learnt have given me the confidence to be able to diagnosis pregnancy and provide follow up USS.	▪ Increased confidence<br>▪ Improved ANC/planning<br>▪ Timely referral
WS1	RM	Y	Yes, better engagement from new antenatal clients.	▪ Increased patient satisfaction & engagement
WS1	RM	Y	Not in antenatal care but have transferred to theatre following confirmed diagnosis of breech (don't do vaginal breech births at our site).	▪ Improved ANC/planning<br>▪ Timely referral<br>▪ Fetal lie

WS2	RM	Y	Mums are very happy about the chance to have a glimpse of their babies. It is helping heaps with the relationship and they start feeling more comfortable earlier and easier to engage in their care.	▪ Increased patient satisfaction & engagement<br>▪ Improved ANC compliance/attendance
WS2	GP	Y	A growth scan in 3rd trimester which lead to early induction for ? macrosomia.	▪ Fetal growth
WS2	GP	Y	Provides me re-assurance that patient is well, and that I have time to manage matters appropriately.	▪ Improved ANC/planning<br>▪ Timely referral
WS2	RM	Y	Yes, it has meant that they only need to go into town (if all is going well) for one-two scans, which for some women is such a relief! I hope that this will decrease our rates of non-attendance, as they will have less trips to take! It has also helped plan their care at a sooner interval, as we know on the spot where this woman is in her pregnancy and what she needs rather than waiting up to a month for a scan and accommodation to be organised.	▪ Improved ANC/planning<br>▪ Timely referral<br>▪ Reduced patient travel<br>▪ Improved ANC compliance/attendance
WS2	RM	Y	A couple of examples: Being able to confidently discuss a client with our sonographer when we required a more detailed scan for a woman in pre-term labour. I was able to provide history, and detail of what we needed to confirm, and why…. so I attended scan and was able to work with sonographer in a more knowledgeable way to obtain results. It is helpful being able to confirm breech position, resulting change of management subsequently, checking a confirmed placenta praevia when a bleed had occurred, while waiting for sonographer to be available, (while managing symptoms) which reassured the anxious mother, as placenta was in place, and we could see a healthy moving baby. There was a partial lift at edge of placenta, which was diagnosed by sonographer an hour later. I attended the scan and was able to have her explain a little more how to identify this. Patient was flown to tertiary hospital. All stable.	▪ Increased confidence<br>▪ Improved ANC/planning<br>▪ Timely referral<br>▪ Reassurance/Reduced patient anxiety<br>▪ Fetal lie<br>▪ Placenta previa
WS3	RN	Y	Yes, there will be an early recognition of problems during antenatal period.	▪ Improved ANC/planning<br>▪ Timely referral
WS3	RM	Y	Dating USS in community meaning accurate EDC and less interventions/time in town for women from remote communities.	▪ Improved ANC/planning<br>▪ Improved estimated due dates<br>▪ Reduced patient travel<br>▪ Estimated gestation/due dates
WS3	RM	Y	Yes, more of the midwives are encompassing USS into their practice and this makes a difference to care coordination.	▪ Improved ANC/planning<br>▪ Timely referral
WS3	RM	Y	Referral to specialists and formal ultrasound has improved with late presentations, confirming fetal position and FHR on difficult abdominal palpation in obese women.	▪ Improved ANC/planning<br>▪ Timely referral<br>▪ Fetal heart<br>▪ Fetal lie
WS3	GP	Y	I can clearly recognise situations where USS is beneficial. I need to further build my skills to be able to trust my findings as reliable.	▪ Lack of confidence
WS3	GP	Y	Probably still too early to make that claim, other than increased patient and partner satisfaction and improved parent-fetal bonding.	▪ Increased patient satisfaction & engagement<br>▪ Partner engagement

WS4	RM	Y	Yes, but still gaining confidence in this area. As a midwife with obstetricians on call and not always present, I have been able to alert to potential risks, (along with other signs)… and confirm the position of a fetus, contributing in making a management decision.	▪ Improved ANC/planning<br>▪ Timely referral<br>▪ Fetal lie
WS4	RM	Y	Whilst still working remote, the timing and frequency of long trips to Alice Springs was improving.	▪ Improved ANC/planning<br>▪ Timely referral<br>▪ Reduced patient travel<br>▪ Economic benefit
WS4	RM	Y	I am able to see when patients are due to have their babies without having to send them to Primary care centre for a dating scan, which is up to 13 hours drive away.	▪ Improved ANC/planning<br>▪ Timely referral<br>▪ Reduced patient travel<br>▪ Economic benefit
WS4	RM	Y	Early detection of fetal demise, correlating with quant HCG for a couple of women, women do come in when the outreach midwife is visiting so they can have a scan just to look. Makes it much easier to do antenatal care when women are keen to come, husbands are also wanting to have a look when culturally this is supposed to be women's business but I think this a good change.	▪ Improved ANC/planning<br>▪ Improved ANC compliance/attendance<br>▪ Increased patient satisfaction & engagement<br>▪ Partner engagement<br>▪ Fetal demise
WS4	RM	Y	More confidence/ability to prompt quicker clinical follow up.	▪ Increased confidence<br>▪ Improved ANC/planning<br>▪ Timely referral
WS4	RM	Y	I have been able to diagnosis a miscarriage and my dating USS are more accurate.	▪ Improved estimated due dates<br>▪ Increased confidence<br>▪ Increased accuracy<br>▪ Improved ANC/planning<br>▪ Timely referral<br>▪ Estimated gestation/due dates<br>▪ Miscarriage

Improvements	Frequency of theme	Applications	Frequency of theme	Barriers	Frequency of theme
▪ Improved ANC & planning	48	▪ Estimated gestation/due dates	22	Equipment	9
▪ Timely referral	48	▪ Fetal lie	20	Need for onsite supervision	3
▪ Improved estimated due dates	21	▪ Fetal viability	7	Opportunity to scan/practice	2
▪ Increased patient satisfaction & engagement	20	▪ Fetal heart	6	Time limits during consultation	2
▪ Increased confidence	18	▪ Fetal demise	4	Socioeconomic	2
▪ Reassurance/Reduced patient anxiety	17	▪ Amniotic fluid Index assessment	4	Lack of confidence	2
▪ Reduced patient travel	14	▪ Fetal biometry	3	Legal/liability concerns	1
▪ Improved ANC compliance/attendance	10	▪ Fetal growth	3	Employer/management support	1
▪ Economic benefit	7	▪ Placental position	3	Cultural	1
▪ Patient education	5	▪ Miscarriage	3	Environmental	1
▪ Improved monitoring	4	▪ Multiple pregnancy	1		
▪ Increased accuracy	3				
▪ Partner engagement	2				
